# A versatile MOF-based trap for heavy metal ion capture and dispersion

**DOI:** 10.1038/s41467-017-02600-2

**Published:** 2018-01-15

**Authors:** Yaguang Peng, Hongliang Huang, Yuxi Zhang, Chufan Kang, Shuangming Chen, Li Song, Dahuan Liu, Chongli Zhong

**Affiliations:** 1 State Key Laboratory of Organic-Inorganic Composites, Beijing University of Chemical Technology, 100029 Beijing, China; 2 State Key Laboratory of Separation Membranes and Membrane Processes, Tianjin Polytechnic University, 300387 Tianjin, China; 3 National Synchrotron Radiation Laboratory, CAS Center for Excellence in Nanoscience, University of Science and Technology of China, 230029 Hefei, China; 4 Beijing Advanced Innovation Center for Soft Matter Science and Engineering, 100029 Beijing, China

## Abstract

Current technologies for removing heavy metal ions are typically metal ion specific. Herein we report the development of a broad-spectrum heavy metal ion trap by incorporation of ethylenediaminetetraacetic acid into a robust metal-organic framework. The capture experiments for a total of 22 heavy metal ions, covering hard, soft, and borderline Lewis metal ions, show that the trap is very effective, with removal efficiencies of >99% for single-component adsorption, multi-component adsorption, or in breakthrough processes. The material can also serve as a host for metal ion loading with arbitrary selections of metal ion amounts/types with a controllable uptake ratio to prepare well-dispersed single or multiple metal catalysts. This is supported by the excellent performance of the prepared Pd^2+^-loaded composite toward the Suzuki coupling reaction. This work proposes a versatile heavy metal ion trap that may find applications in the fields of separation and catalysis.

## Abstract (web summary)

Heavy metal removal from polluted water is of global significance, but current adsorbents are limited by their specificity towards certain metals. Here the authors incorporate a non-specific and strong chelating group into a robust metal-organic framework and demonstrate its versatility for heavy metal adsorption.

## Introduction

Water quality and scarcity are of growing global concern in the twenty-first century^1, 2^, and thus it is of great importance to remove heavy metal ions from polluted water^3, 4^. Among the various technologies that exist for heavy metal ion removal from aqueous solution, adsorption holds considerable promise due to its simplicity and cost efficiency^5–7^. At present, diverse adsorbents have been developed and investigated for their removal performance^8–11^. However, most adsorbents, such as activated carbons^12^, clays,^13^ and zeolites^14^, show weak binding affinities toward metal ions. To achieve high efficiency, complex functional groups have been introduced to these porous materials to obtain improved metal uptake capacity^15–18^. Despite these progresses, the existing adsorbents still suffer from one main problem: specificity in heavy metal capture, that is, although they show good adsorption performance for one or several given heavy metal ions, they are poorly effective in disposing of other metal ions^16, 19^. In fact, heavy metal species originating from industrial activities such as mining, electroplating, fabrication of barriers, and smelting, are always multifarious and complex^20, 21^. This limitation associated with existing adsorbents can be explained from the prospective of hard and soft acids and bases^22^. On the one hand, the hard and soft characters of functional ligands implement obvious specificity toward different types (hard acid, soft acid, and borderline acid) of heavy metal ions for most existing sorbents. On the other hand, most functional ligands, especially monodentate ligands, have weak coordinating abilities, which leads to the fact that adsorbents functionalized with hard or soft bases can hardly perform efficiently for various hard or soft heavy metal ions. In addition, existing adsorbents still face other challenges such as low density and improper distribution of functional groups on sorbents, which may result in low capacity and moderate affinity for heavy metal species. As a result, development of broad-spectrum adsorbents with well-distributed high-density adsorption sites as well as strong binding affinity toward various heavy metal ions is highly needed.

To tackle this need, we propose the creation of a broad-spectrum heavy metal ion trap (BS-HMT) possessing the following features: strong metal chelating groups thus implementing non-specific high affinity toward different heavy metal species, high loading amount and atomic-level dispersion of chelating groups thereby affording ordered and high-density accessible binding sites, exceptional water/chemical stability facilitating multiple use and regeneration, and high efficiency in both batch adsorption and breakthrough processes offering potential possibility for practical applications. Thus such a BS-HMT could serve as an efficient adsorbent for multifarious and complex metal ion scavenging. On the other hand, the obtained BS-HMT might also serve as a platform for the controlled incorporation of metal ions with good dispersion and may thus find application in the field of catalysis, providing a new strategy for the fabrication of atomic-level-distributed metal catalysts.

To fulfill this goal, a strong non-specific chelating group is needed, for which ethylenediaminetetraacetic acid (EDTA), owing to its six binding sites: four hard carboxyl and two relatively softer tertiary amine groups, is a good candidate. The large coordination number and strong binding affinity associating with both hard and soft characters mean that EDTA can bind to various heavy metal species^23^. However, the resulting metal–ligand complex is always water soluble, limiting its application as an adsorbent. Immobilization of EDTA on porous materials could facilitate separation and recovery for reuse while maintaining active sites for heavy metal ion capture. On the other hand, metal-organic frameworks (MOFs), a relatively new type of porous crystalline materials, are receiving increasing attention^24–29^. Their nanoscale feature of facile postsynthetic modification makes them ideal host materials for active guest species^30–33^, stimulating us to try to realize the BS-HMT concept based on the incorporation of EDTA into a suitable MOF.

Herein, as a proof-of-concept experiment, we demonstrate that such a BS-HMT can be achieved by grafting EDTA to a highly robust MOF (MOF-808)^34, 35^. The resulting BS-HMT, MOF-808-EDTA, shows good single-component adsorption performance for 22 metal ions, including soft acids, hard acids, and borderline acids, and the removal efficiency is >99% for all cases. More importantly, the multi-component experiments show that it also works well for simultaneously treating diverse heavy metal species with ultrahigh removal efficiency in both batch adsorption and breakthrough processes. Particularly, in the latter case the terminal concentration of all the heavy metal ions tested was reduced from 5 ppm to extremely low levels (0.01–1.9 ppb), with a reduction of three to five orders of magnitude, which are much lower than the acceptable limits in drinking water standards of the World Health Organization (WHO). In addition, this MOF-based HMT can be readily regenerated without significant loss of metal uptake capacity. The results also show that the BS-HMT can serve as a new strategy for loading metal ions with arbitrary selections of metal ion types and uptake ratios to prepare well-dispersed single or multiple metal catalysts, as supported by the excellent performance of the prepared Pd^2+^@BS-HMT toward the Suzuki coupling reaction.

## Results

### Preparation of the BS-HMT

**Fig. 1:**
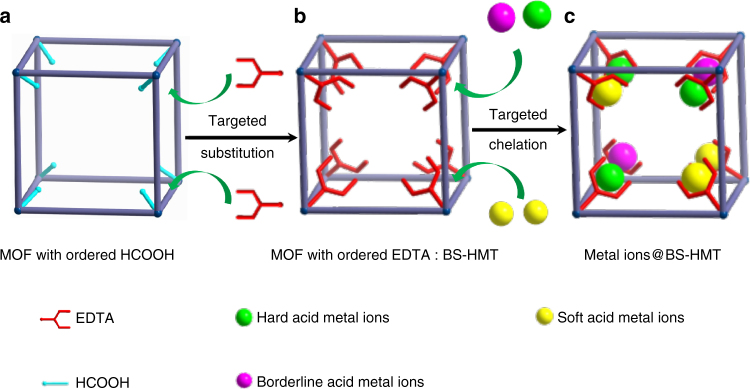
Schematic illustration of the BS-HMT concept. **a** The ordered HCOOH in MOF-808 can be substituted by EDTA to form **b** MOF-808 with ordered EDTA, which can be used as **c** a BS-HMT for metal ion capture

**Fig. 2:**
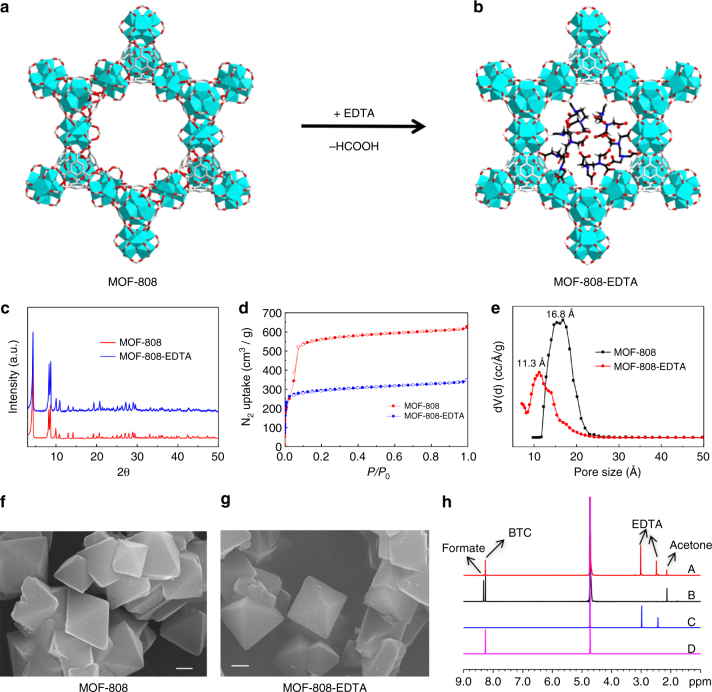
Characterization and illustration of MOF-808 and MOF-808-EDTA. **a**, **b** Schematic illustration of the structures of the two MOFs. **c** PXRD patterns. **d** N_2_ adsorption-desorption isotherms. **e** Pore size distributions. **f**, **g** SEM images (Scale bar, 500 nm). **h** ^1^H NMR spectra of (A) alkaline-digested MOF-808-EDTA, (B) alkaline-digested MOF-808, (C) EDTA-2Na, and (D) H_3_BTC in KOH/D_2_O solution

A schematic illustration of the construction of the BS-HMT is shown in Fig. 1, in which the MOF-based HMT was formed by functionalizing MOF-808 with EDTA through solvent-assistant linker exchange method^36^ (Fig. 2a, b). MOF-808 has a 6,3-connected three-dimensional framework with an overall *spn* topology and two kinds of cages^34^. The tetrahedral cages are constructed by inorganic secondary building units (SBUs) at the vertices and the benzene-1,3,5-tricarboxylate (BTC) linkers at the faces. The large adamantane cages are formed by the tetrahedral cages act as vertices. This Zr-MOF was chosen as a postsynthetic modification platform for the following reasons: high surface area and large cavity size, exceptional high water/chemical stability, the formate groups on Zr_6_ cluster can be targeted substituted by other ligands, and it is biocompatible, degradable, and free of contamination^37^, having widely potential applications in effluent treatment. The EDTA-functionalized MOF-808 (named as MOF-808-EDTA) was achieved by submerging MOF powders in ethylenediaminetetraacetic acid disodium salt (EDTA-2Na) aqueous solution, which was regarded as a convenient, low-cost, and green synthesis process.

Figure 2c shows the powder X-ray diffraction (PXRD) patterns of MOF-808-EDTA and pristine MOF-808. It is apparent that the related peaks of these two MOFs are closely matched, confirming that the crystal structure of material remains intact after EDTA modification. Nitrogen adsorption–desorption isotherms measured at 77 K illustrate that the introduction of EDTA molecules into the framework leads to a decrease in the Brunauer–Emmett–Teller surface area from 2424 m^2^ g^−1^ to 1173 m^2^ g^−1^ (Fig. 2d). The pore size distribution reveals a small reduction after EDTA modification from 16.8 Å to 11.3 Å (Fig. 2e). It needs to note that such cavity size in MOF-808-EDTA (11.3 Å) is still large enough for efficient mass transfer during metal ion capture. Scanning electron microscopy indicates that MOF-808-EDTA possesses octahedral morphology, with no significant morphology change being observed after modification (Fig. 2f, g). Thermogravimetric analysis reveals that MOF-808-EDTA could maintain thermal stability up to 250 °C, which is consistent with pristine MOF-808 (Supplementary Fig. 1). The successful grafting of EDTA onto MOF-808 can also be confirmed by Fourier transform infrared (FT-IR) spectroscopy, X-ray photoelectron spectroscopy (XPS), ^1^H nuclear magnetic resonance (NMR) spectroscopy, and elemental analysis. The FT-IR spectra of MOF-808-EDTA show that there is a new peak at 1214 cm^−1^ compared with pristine MOF-808, which can be assigned to the C-N stretching band (Supplementary Fig. 2)^38^. XPS spectra of MOF-808-EDTA in Supplementary Fig. 3 indicate the appearance of nitrogen signal at the binding energy of 399.2 eV, which is attributed to the N 1s of EDTA. Figure 2h shows ^1^H NMR spectra of MOF-808 and MOF-808-EDTA after dissolving the samples in KOH/D_2_O solution. It is obvious that the chemical shift at 8.3 ppm for the hydrogen of formate group in MOF-808-EDTA has disappeared and two additional chemical shifts at 2.5 and 3.0 ppm corresponding to different hydrogen signals of -CH_2_- in EDTA are present, indicating that the formate ligands on Zr_6_ clusters are almost entirely substituted by EDTA molecules. The two relevant signals of the incorporated EDTA were then integrated against that of BTC ligand, resulting in peak ratios of 6:16 and 6:8, respectively, indicating that about two bound EDTA were observed per Zr_6_ SBU in the frameworks of MOF-808-EDTA (Supplementary Fig. 4). Elemental analysis reveals a nitrogen content of 3.16% for MOF-808-EDTA. By assuming that all the N atoms come from loaded EDTA molecules, the EDTA content in MOF was estimated to be as high as 33%. All of these observations demonstrate that EDTA is successfully introduced into the pore structure of MOF-808 with high loading. To prove that EDTA is actually attached to the framework of MOF-808 instead of being adsorbed in the MOF channels, the MOF-808-EDTA was first analyzed by XPS spectra. As shown in Supplementary Fig. 5, the Zr 3d core lever exhibits a significant shift after modification, indicating that the EDTA molecules are grafted on the Zr_6_ clusters of MOF-808. To further confirm this, the EDTA-modified samples were washed with water repeatedly to check whether EDTA will leak out from MOF solid. Such samples were then subjected to ^1^H NMR and elemental analysis. As shown in Supplementary Fig. 6, the ^1^H NMR spectrum of water-washed MOF-808-EDTA is consistent with that of fresh one. By integrating the related signals of EDTA against that of BTC ligand, the EDTA-loading amount shows almost no change after washing with water for several times, indicating that EDTA is actually grafted on the framework instead of being adsorbed in MOF channels. This result is also supported by the similar nitrogen contents (Supplementary Table 1) by elemental analysis for the solid samples before and after washing with water repeatedly. Based on above experimental results, the formulate of MOF-808-EDTA can be denoted as Zr_6_O_x_(OH)_y_(C_9_H_3_O_6_)_2_(EDTA)_2_(HCOO)_0.24_. For MOF-808, (*x* + *y*) is always equal to 8 since there are 8 O atoms around the Zr_6_ cluster. Based on the theory of conservation of charge, the formulate of MOF-808-EDTA is eventually denoted as Zr_6_O_7.76_(OH)_0.24_(C_9_H_3_O_6_)_2_(EDTA)_2_(HCOO)_0.24_. To identify the local coordination environments of EDTA in MOF-808-EDTA, the X-ray absorption near-edge structure for the Zr_6_ cluster in MOF-808 and MOF-808-EDTA are shown in Supplementary Fig. 7a. Clearly, the position of the Zr K edge and the corresponding shapes are almost the same for both the samples, indicating similarities in the Zr^4+^ coordination environment. A very slight shift of absorption edge in Supplementary Fig. 7a indicates that Zr^4+^ gets electron when formate is replaced by EDTA in MOF-808. To get more detailed structural information about the local Zr^4+^ environment, the EXAFS data were collected, and subsequently the Fourier-transformed EXAFS spectra were obtained, as shown in Supplementary Fig. 7b. The coordination number of O around Zr^4+^ in MOF-808-EDTA was obviously changed. For the longer Zr-O bonds, the coordination number of O around Zr^4+^ was enhanced, while for the shorter Zr-O bonds, the coordination number was reduced. These EXAFS results suggested the EDTA was coordinated with Zr_6_ cluster by single -COOH group of EDTA. Therefore, the remained five binding sites of EDTA can be used as a working group for metal ion capture.

### Capture performance for single-component systems

**Fig. 3:**
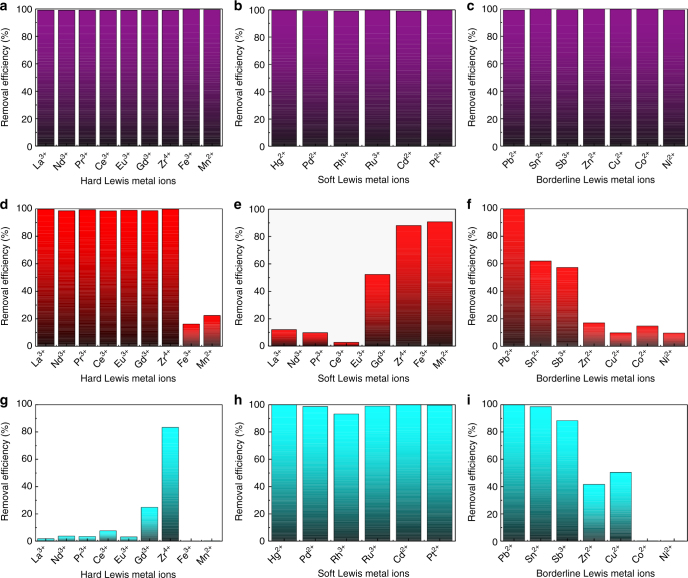
Removal efficiency of heavy metal ion in single-component systems. The removal efficiency of hard Lewis metal ions, soft Lewis metal ions, and borderline Lewis metal ions for **a**–**c** MOF-808-EDTA, **d**–**f** MOF-808-OX, and **g**–**i** MOF-808-TGA

To evaluate the effectiveness of MOF-808-EDTA as a general HMT, a total of 22 different kinds of heavy metal ions were selected as target ions to capture, which can be divided into three types: soft acids, hard acids, and borderline acids. As shown in Fig. 3a–c, MOF-808-EDTA performs excellently, and for all the tested metal ions, the removal efficiencies are >99%. Owing to the fact that EDTA-modified material has both hard and relative softer sites together with high coordination number, MOF-808-EDTA exhibits ultrahigh removal efficiency toward all three types of heavy metal ions, while most exiting adsorbents, which exhibits obvious specificity, can only trap those that belong to one or two types of heavy metal ions.

To confirm this hypothesis, control experiments were conducted. Two functional ligands, oxalic acid (OX) and thioglycollic acid (TGA), were introduced into the framework of MOF-808 and two new MOFs (MOF-808-OX and MOF-808-TGA) bearing hard (-COOH) and soft (-SH) ligands were prepared, respectively (Supplementary Figs 8–19). As we expected, MOF-808-OX shows high removal efficiency for hard metal ions compared with MOF-808-TGA, while the latter can retain high removal efficiency toward soft metal ions and barely adsorb hard ones (Fig. 3d–i). Notably, both MOF-808-OX and MOF-808-TGA can hardly adsorb all three types of the tested metal ions. Even for individual hard or soft metal ions, these two MOFs still show inferior performance compared with MOF-808-EDTA. These results well illustrate that MOF-808-EDTA is a BS-HMT.

To investigate the efficiency and capacity of metal ions uptake in MOF-808-EDTA, the two key metrics for performance assessment, adsorption kinetics, and isotherms were further measured. For convenience, La^3+^, Hg^2+^, and Pb^2+^ were selected as an example that belong to hard acid, soft acid, and borderline acid, respectively. As shown in Supplementary Fig. 20, extremely fast adsorption kinetics was observed. The remove efficiency can exceed 90% in 5 min and reach 99% after equilibrium for all the three ions. The high-rate performance can be ascribed to its surface area and pore size, which are large enough to facilitate the diffusion of metal ions to the EDTA sites of MOF-808. The experimental data are fitted well with pseudo-second-order model (Supplementary Note 1), which suggested that the rate-determined step for the adsorption of metal ions onto MOF-808-EDTA is mainly chemical^39^. Supplementary Fig. 21 shows the adsorption isotherms of MOF-808-EDTA toward the three metal ions. By fitting with Langmuir model^40^ (Supplementary Note 2), the saturated adsorption capacities of MOF-808-EDTA for La^3+^, Hg^2+^, and Pb^2+^ are calculated to be 205 mg g^−1^, 592 mg g^−1^, and 313 mg g^−1^ respectively, which are higher than various porous materials^41–47^. To further demonstrate the advantage of our MOF-based material, we also compare the capture performance of MOF-808-EDTA with other EDTA-modified adsorbents, including both organic and inorganic materials. As shown in Supplementary Fig. 22 and Supplementary Tables 2–5, MOF-808-EDTA performs best as a whole, particularly for heavy metal ion capture, due to the well-dispersed ordered structure of EDTA in the MOF.

**Fig. 4:**
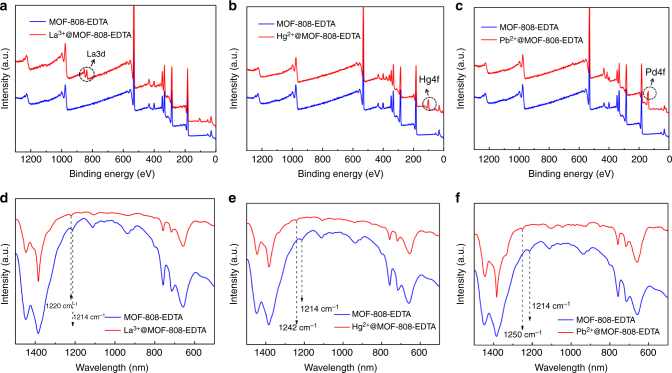
Characterization of MOF-808-EDTA before and after metal ion loading. **a**–**c** Wide-scan XPS spectra and **d**–**f** FT-IR spectra of MOF-808-EDTA before and after La^3+^, Hg^2+^, and Pb^2+^ loading

The excellent performance of MOF-808-EDTA as BS-HMT can be traceable to the strong chelation between metal ions and EDTA in MOF-808-EDTA, which were verified by XPS and FT-IR. Here, La^3+^, Hg^2+^, and Pb^2+^ were selected as examples. The wide-scan XPS spectra shown in Fig. 4a–c indicate that the metal ions are adsorbed in the pores of MOF-808-EDTA solid. The N 1s core level is shifted to higher binding energy upon metal loading (399.8 eV, 399.5 eV, and 399.9 eV for La^3+^@MOF-808-EDTA, Hg^2+^@MOF-808-EDTA, and Pb^2+^@MOF-808-EDTA, respectively) compared with the as-synthesis analog (399.2 eV), indicating that the valence of N in EDTA is changed because of the interaction with the guest metal ions (Supplementary Figs. 23–25). Moreover, IR spectra reveal an obvious shift of C-N vibration mode in MOF-808-EDTA from 1214 cm^−1^ to 1220 cm^−1^, 1242 cm^−1^, and 1250 cm^−1^ for La^3+^-, Hg^2+^-, and Pb^2+^-loaded MOF-808-EDTA, respectively (Fig. 4d–f). These results suggest the strong interaction between three types of heavy metal ions (soft acid, hard acid, and borderline acid) and EDTA functional groups' grafting on the framework, resulting in the chelate complex after adsorption. In addition, to prove that EDTA binds strongly to Zr species and will not leak out from MOF SBU, the MOF-808-EDTA samples were immersed in metal ions' aqueous solution with a concentration of 1000 ppm. Here, La^3+^, Pb^2+^, and Hg^2+^ were selected as examples. The solids obtained by filtration were then subjected to ^1^H NMR spectra. As shown in Supplementary Fig. 26, MOF-808-EDTA exhibits similar ^1^H NMR spectra before and after metal ion loading. By integrating the related signals of EDTA against that of BTC ligand, it is apparent that the amount of EDTA in MOF-808-EDTA has no obvious change after immersion in La^3+^, Hg^2+^, and Pb^2+^ aqueous solution with high concentration, indicating that little EDTA molecules will leak out from Zr_6_ clusters into the solution, even in high concentration of metal ions.

### Capture performance for multi-component systems

**Fig. 5:**
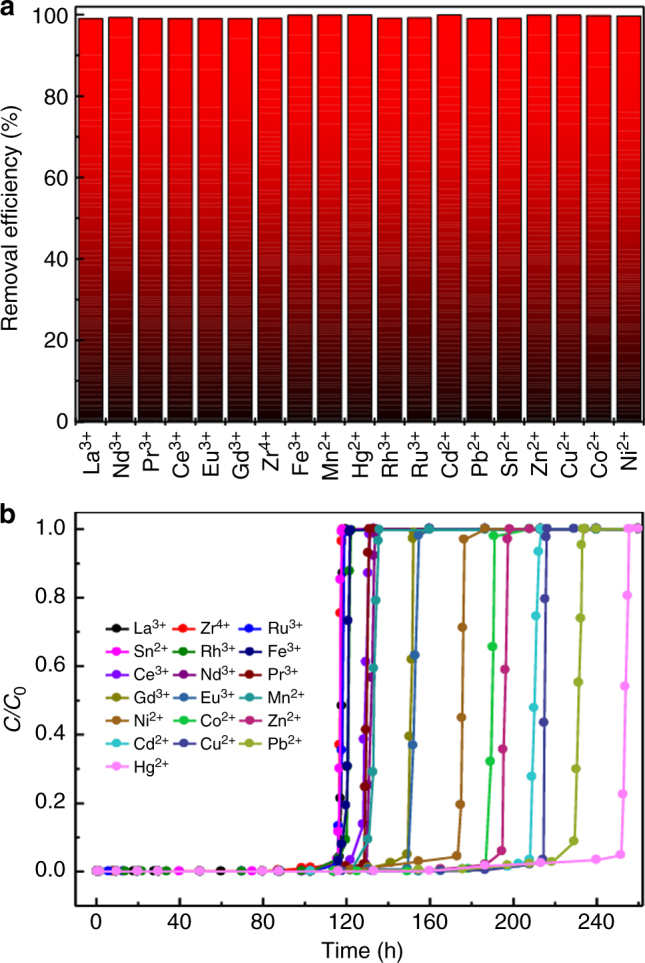
Capture performance of MOF-808-EDTA in multi-component systems. **a** Simultaneous removal efficiency for 19 metal ions in batch adsorption. **b** Breakthrough curves in the fixed bed adsorption

Considering that some polluted water may contain multiple metal contaminants, we further investigated the efficiency of the BS-HMT for removing diverse heavy metal ions simultaneously, in which both batch adsorption and breakthrough processes were considered. Here a solution containing 19 kinds of metal ions was used for evaluation (since the salts of Pt^2+^, Pd^2+^, and Sb^3+^ cannot dissolve in water directly, they are excluded in the multi-component adsorption experiments). As shown in Fig. 5a, all the metal ions have been effectively captured with a removal efficiency of >99% in the batch adsorption process, the breakthrough experiment with the above solution flowing over a packed bed of MOF-808-EDTA solid with a flow rate of 0.25 ml min^−1^ at room temperature shows that the terminal concentrations of all the tested metal ions were reduced from 5 ppm to extremely low level (0.01–1.9 ppb), with a reduction of three to five orders of magnitude. For Cd^2+^, Cu^2+^, Sn^2+^, Pb^2+^, Mn^2+^, Hg^2+^, and Ni^2+^, the terminal concentrations are all much lower than the acceptable limits in drinking water standards of WHO (Fig. 5b, for details, see Supplementary Fig. 27) (for other metal ions considered in this work, there are no specific guide values in WHO standards). All the breakthrough curves increase sharply, indicating fast mass transfer and sorption kinetics. The exciting results well indicate that MOF-808-EDTA is a promising material for purifying multiple metal contaminants simultaneously that may find practical use.

Cycle performance of porous material is also crucial for application. Notably, the metal ions-loaded MOF-808-EDTA can be regenerated by being washed using a high concentration of EDTA-2Na solution for several times. Supplementary Fig. 28 shows that the color of MOF-808-EDTA changes from white to blue after Cu^2+^ ions' loading and returns to white upon rinsing with EDTA-2Na solution. The regenerated MOF-808-EDTA was then subjected to the next round metal ion removal, for which La^3+^ (hard acid), Hg^2+^ (soft acid), and Pb^2+^ (borderline acid) were taken as typical examples. As shown in Supplementary Fig. 29, the adsorption capacity of MOF-808-EDTA can be retained over 90% for all the tested metal ions after four consecutive cycles. In addition, we also investigated the stability of MOF-808-EDTA toward all the 22 metal ions. As shown in Supplementary Fig. 30, the comparability of the related peak patterns of MOF-808-EDTA before and after heavy metal ion removal indicates the framework retains intact, and no apparent collapse occurs during sorption and regeneration processes.

### Dispersity of metal ions in the BS-HMT

The BS-HMT also holds great potential for use as solid support for controlled loading/dispersion of metal ions at the atomic level, since the ordered atomic-level-dispersed EDTA has strong chelation toward heavy metal ions and each EDTA molecule can only binds one metal ion. This unique feature of the proposed BS-HMT makes it versatile that may incorporate one kind or several kinds of metal ions with atomic-level dispersion, showing great potential in other fields, such as catalysis (Fig. 1c).

**Fig. 6:**
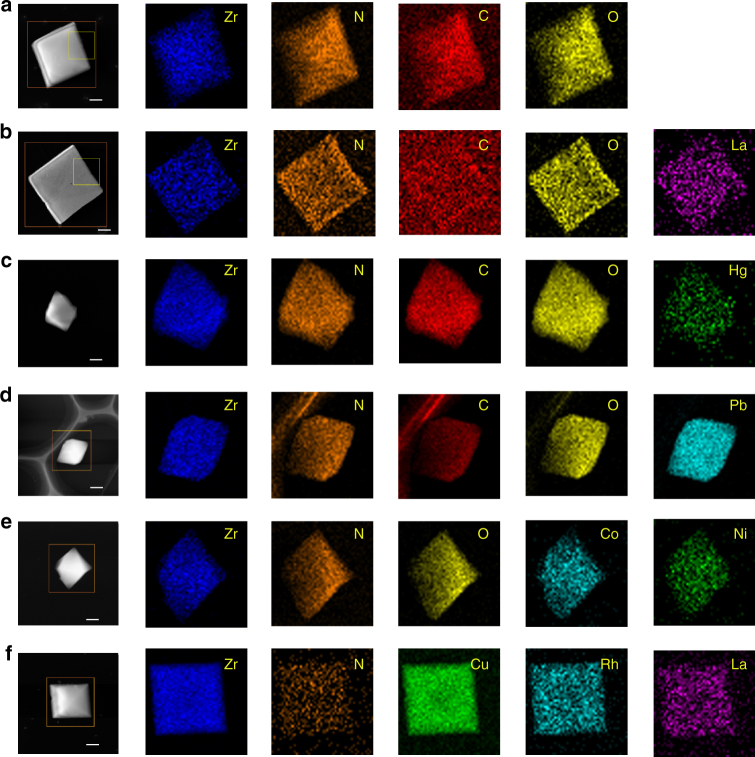
Dispersity of metal ions in MOF-808-EDTA. STEM-HAADF images (scale bar, 100 nm) and the corresponding elemental maps for **a** MOF-808-EDTA, **b**–**d** single-metal systems (MOF-808-EDTA with loaded La^3+^, Hg^2+^, and Pb^2+^, respectively), **e** binary system (MOF-808-EDTA with loaded Co^2+^ and Ni^2+^), and **f** ternary system (MOF-808-EDTA with loaded Cu^2+^, Rh^3+^and Ru^3+^)

To verify the above hypothesis, we further analyzed the heavy metal ion-adsorbed MOF-808-EDTA samples by high-angle annular dark-field scanning transmission electron microscopy (HAADF-STEM). First, the single-metal ion-loaded samples for La^3+^ (hard acid), Hg^2+^ (soft acid), and Pb^2+^ (borderline acid) were analyzed. As shown in Fig. 6a, elemental mapping using HAADF-STEM demonstrates nitrogen disperses uniformly within the crystal samples of MOF-808-EDTA, suggesting the well dispersion of EDTA which can serve as specific metal attachment sites for precise control of the metal ions' distribution within the frameworks. Figure 6b–d shows the elemental mappings of single-metal-loaded MOF-808-EDTA. As expected, La^3+^, Hg^2+^, and Pb^2+^ uniformly disperse in the framework giving good support to our hypothesis. Second, we further analyzed the samples with two or three metal ions loaded simultaneously. Figure 6e shows the results for the two-metal sample of Co^2+^/Ni^2+^@MOF-808-EDTA. Obviously, similar to the one-metal case, Co^2+^ and Ni^2+^ disperse uniformly in the framework of MOF-808-EDTA, which is also the case for the three-metal sample of La^3+^/Rh^3+^/Cu^2+^ @MOF-808-EDTA (Fig. 6f). The last case deals with three types of metal ions simultaneously (La^3+^(hard acid), Rh^3+^ (soft acid), and Cu^2+^ (borderline acid)), highlighting the feature of the proposed versatile BS-HMT. In addition, the ratio of the metal ions loaded can be tuned easily: as shown in Supplementary Fig. 31, the ratio of the metal ions loaded in the BS-HMT shows a strong correlation with the initial ratio of the metal ions in solution.

One of the main applications of the incorporation of metals/metal ions to porous materials is to prepare supported metal catalysts, in which a proper dispersion of active sites is crucial. To highlight the applicability of the proposed BS-HMT methodology for preparing catalysts, Suzuki coupling reaction was chosen as a proof-of-concept example. As shown in Supplementary Table 6, Pd^2+^@MOF-808-EDTA exhibits excellent performance for this reaction, with both conversion and selectivity being over 99%, suggesting the great potential of MOF-808-EDTA as solid support in preparation of metal catalysts with good dispersion. To verify whether the EDTA grafted on the framework of MOF-808 will leak out during the catalytic reaction, the Pd^2+^@MOF-808-EDTA solid samples before and after Suzuki coupling reaction were analyzed by ^1^H NMR spectra. As shown in Supplementary Fig. 32, by integrating related signals of BTC ligand against that of EDTA, the peak ratios of BTC ligand with EDTA in MOF were changed from 6:15.91 and 6:7.96 to 6:13.32 and 6:6.65, respectively after the catalytic reaction, indicating a slight decrease (~16%) of the EDTA amount in MOF solid. Besides, the peak at 8.3 ppm corresponding to the signal of formate was observed to enhance significantly. This can be explained that a small amount of solvent dimethylformamide (DMF) decomposes into formate at high temperature during the catalytic reaction. Extra formates may bind to Zr_6_ clusters of MOF-808 again, resulting in the slight decrease of the EDTA-loading amount in MOF and the enhancement of formate in the catalyst.

In summary, existing adsorbents normally show specificity in heavy metal capture, which largely limits their application in real wastewater treatment that contains multifarious and unpredictable heavy metal species. To tackle this problem, we proposed the concept of a BS-HMT by grafting EDTA into the pores of a robust MOF. As a proof-of-concept experiment, MOF-808-EDTA was prepared. The results for a total of 22 kinds of metal ions covering soft acids, hard acids, and borderline acids show that it can non-specifically capture all the metal ions with removal efficiencies of >99%. Notably, their efficiency remains excellent in treating multiple heavy metal ions simultaneously. The ultrahigh removal efficiency for multifarious heavy metal species in both one- and multi-component systems sets a new benchmark for heavy metal adsorbent materials. Breakthrough test reveals that the terminal concentration of the mixture containing 19 kinds of metal ions was reduced from 5 ppm to extremely low levels (0.01–1.9 ppb), with a reduction of three to five orders of magnitude, much lower than the acceptable limits in drinking water standards of the WHO, showing good potential in industrial application. Furthermore, the BS-HMT can also be used as a strategy for the incorporation of one kind or a combination of several kinds of metals with atomic-level uniform dispersion, which is of significance for the preparation of single/multiple-metal catalysts. Therefore, the proposed BS-HMT is versatile and provides a platform for metal capture and loading/dispersion.

## Methods

### Characterizations

PXRD data were measured on D8 Advanced X diffractometer with Cu Kα radiation (*λ* = 1.5406 Å) at a step size of 0.02°. The nitrogen adsorption–desorption isotherms of MOF-808-EDTA were examined by using Autosorb-IQ-MP (Quantachrome Instruments) at 77 K. The morphologies of the prepared MOFs were obtained by a Hitachi S-4700 scanning electron microscope. ^1^H NMR spectra were measured on a Bruker Fourier 600M spectrometer. Thermogravimetric curves were obtained on a thermal gravimetric analysis with a heating rate of 10 °C/min in air. The IR spectra were determined with a Nicolet 6700 FTIR spectrophotometer. The elemental analysis was measured by vario EL cube (Elementar). A Tecnai G2 F20 transmission electron microscope (FEI) equipped with an energy-dispersive X-ray spectrometer system and HAADF detector was used at 200 kV for high-resolution electron microscopy imaging and HAADF imaging. The results of XPS were obtained by using an ESCALAB 250 X-ray photoelectron spectroscopy with Al Kα X-ray as the excitation source. The concentration of metal ions in aqueous solution was determined by an inductively coupled plasma-mass spectrometry (ICP-MS). Zr K-edge X-ray absorption spectroscopy were collected in beamline 1W1B in Beijing Synchrotron Radiation Facility (BSRF). The X-ray was monochromatized by a double-crystal Si (111) monochromator for BSRF. The energy was calibrated using a zirconium metal foil for the Zr K-edge. The monochromator was detuned to reject higher harmonics. The acquired EXAFS data were processed according to the standard procedures using the WinXAS3.1 program^48^.

### Synthesis of MOF-808-EDTA

MOF-808(Zr) was prepared according to previous literature^34, 35^. Then about 0.100 g of activated MOF-808(Zr) was added to 1.860 g of EDTA-2Na in a solution containing 50 ml of water. The contents were placed in a 100 ml screw-capped glass jar, which was heated to 60 °C for 24 h. A white precipitate was obtained by filtration and washed with water for several times to remove unreacted EDTA. Subsequently, the solid sample was immersed in fresh acetone to exchange water in the pores of MOF-808-EDTA and this procedure was repeated for several times. The solid was then dried at 60 °C overnight under vacuum condition. Elemental Analysis: C: 25.61%; H: 2.26%; N: 3.16%.

### Synthesis of other materials

The detail of synthesizing other materials can be found in Supplementary Methods.

### Single-component batch adsorption experiments

A total of 22 heavy metal ions (La^3+^, Nd^3+^, Pr^3+^, Ce^3+^, Eu^3+^, Gd^3+^, Zr^4+^, Fe^3+^, Mn^2+^, Hg^2+^, Rh^3+^, Ru^3+^, Cd^2+^, Pb^2+^, Sn^2+^, Zn^2+^, Cu^2+^, Co^2+^, Ni^2+^, Pt^2+^, Pd^2+^, and Sb^3+)^ containing hard acids, soft acids, and borderline acids were selected as target ions. The stock solution of heavy metal ions was prepared by dissolving relevant metal salt in water directly except for Pt^2+^, Pd^2+^, and Sb^3+^. Considering Pt(NO_3_)_2_, PdCl_2_, and SbCl_3_ cannot dissolve in water directly, corresponding stock solution was prepared in acidic condition (pH ≈ 2). In a typical adsorption, MOF-808-EDTA (0.010 g) was added into a glass bottle containing 10 ml stock solution of heavy metal ions with the concentration of 10 ppm. The mixture was kept at room temperature for 24 h and filtered through a 0.22 µm membrane filter for all samples. Subsequently, the filtrates were measured by using ICP-MS to determine the residual metal content.

### Adsorption kinetics measurement

MOF-808-EDTA sample (0.010 g) was added into a glass bottle containing 10 ml stock solution of heavy metal ions with the concentration of 10 ppm. The mixture was kept at room temperature for 24 h. During the adsorption period, the mixture was filtered at intervals through a 0.22 µm membrane filter for all samples. Subsequently, the filtrates were measured by using ICP-MS to determine the residual metal content.

### Adsorption isotherm measurement

MOF-808-EDTA sample (0.010 g) was added into a glass bottle containing 10 ml stock solution of heavy metal ions with different concentrations. The mixture was kept at room temperature for 24 h and filtered through a 0.22 µm membrane filter for all samples. Subsequently, the filtrates were measured by using ICP-MS to determine the residual metal content.

### Multi-component batch adsorption experiments

MOF-808-EDTA sample (0.095 g) was added into a glass bottle containing a 10 ml stock solution of 19 heavy metal ions (since the salts of Pt^2+^, Pd^2+^, and Sb^3+^ cannot dissolve in water directly, they are excluded in the multi-component adsorption experiments) with each metal concentration of 5 ppm. The mixture was kept at room temperature for 24 h and filtered through a 0.22 µm membrane filter. Subsequently, the filtrates were measured by using ICP-MS to determine the residual metal content.

### Breakthrough experiment

MOF-808-EDTA sample (about 3.0 g) was packed into a column with inner diameter of ~10 mm. The packed sample length is about 6 cm. An aqueous solution containing 19 kinds of heavy metal ions (since the salts of Pt^2+^, Pd^2+^, and Sb^3+^ cannot dissolve in water directly, they are excluded in the multi-component adsorption experiments) was then passed through the column with a flow of 0.25 mlmin^−1^ at room temperature, and the filtrates were measured by using ICP-MS to determine the residual metal content.

### Suzuki coupling reaction by Pd^2+^@MOF-808-EDTA

Iodobenzene or its analogs with different functional groups (0.1 mmol), phenylboronic acid (0.6 mmol), and K_2_CO_3_ (1 mmol) as base were dissolved in 10 ml DMF. Subsequently, Pd^2+^@MOF-808-EDTA (0.025 g) was added to the mixture. The catalytic reaction was conducted at 80 °C for 3 h. The final product was analyzed by GC (FuLi-9790II) with a flame-ionization detector using a weak polarity column (AT.SE-54).

### Data availability

The data that support the findings of this study are available from the corresponding author.

## Electronic supplementary material

Supplementary Information
