# Supplementary material for: A versatile MOF-based trap for heavy metal ion capture and dispersion
Source: Nat Commun. 2018 Jan 15;9:187. doi: 10.1038/s41467-017-02600-2 (PMC5768720; doi:10.1038/s41467-017-02600-2)
Supplement: Supplementary file 1 — Supplementary Information [file 41467_2017_2600_MOESM1_ESM.pdf]

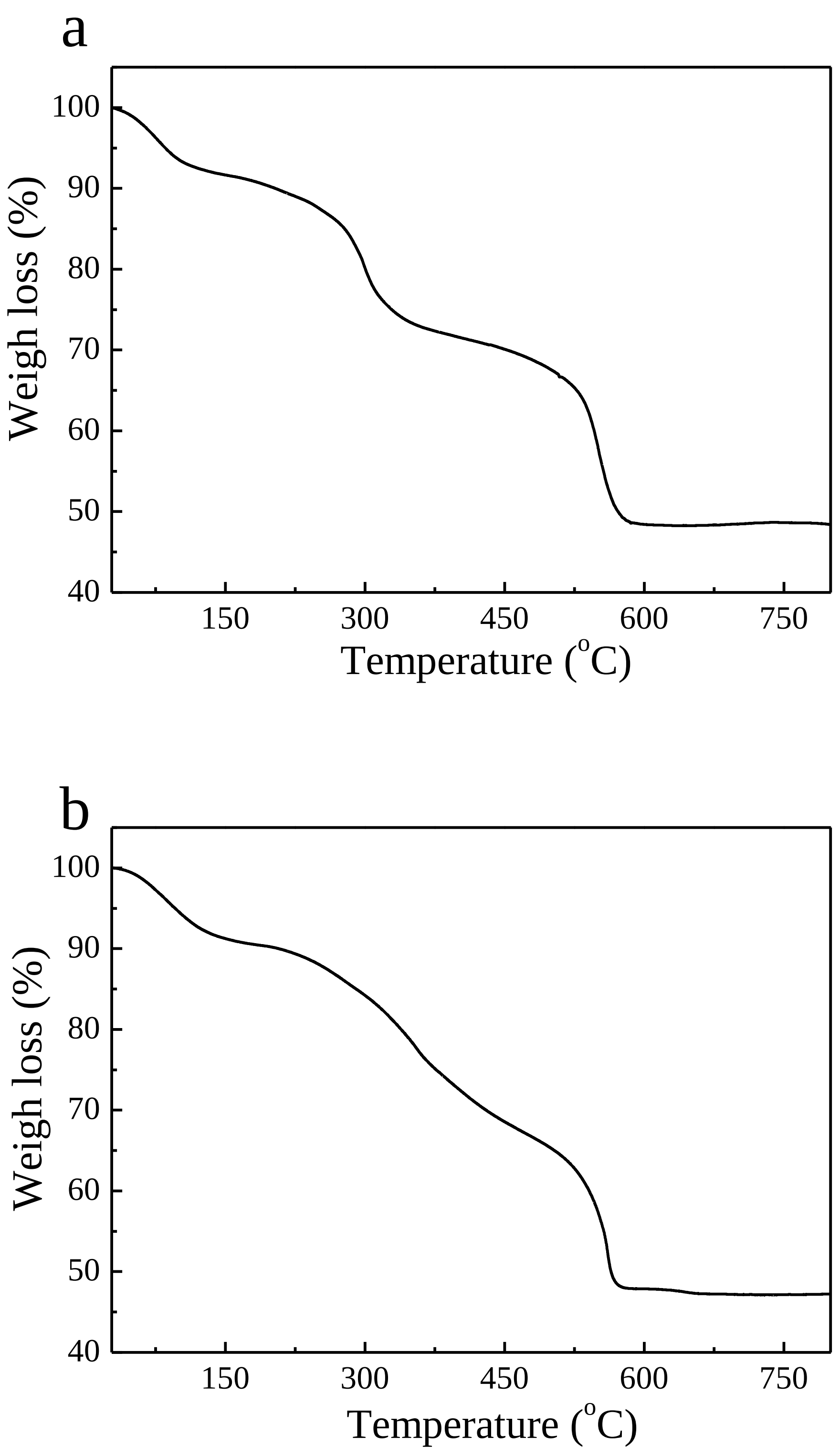

Supplementary Figure 1 | TGA plots of (a) MOF-808 and (b) MOF-808-EDTA.

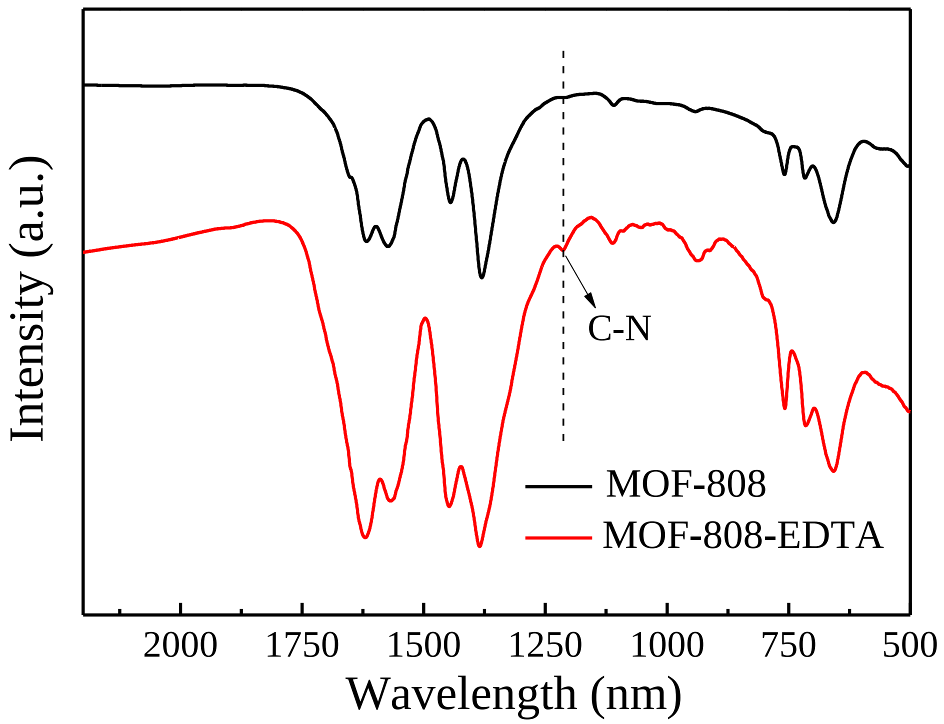

Supplementary Figure 2 | IR spectra of MOF-808 and MOF-808-EDTA.

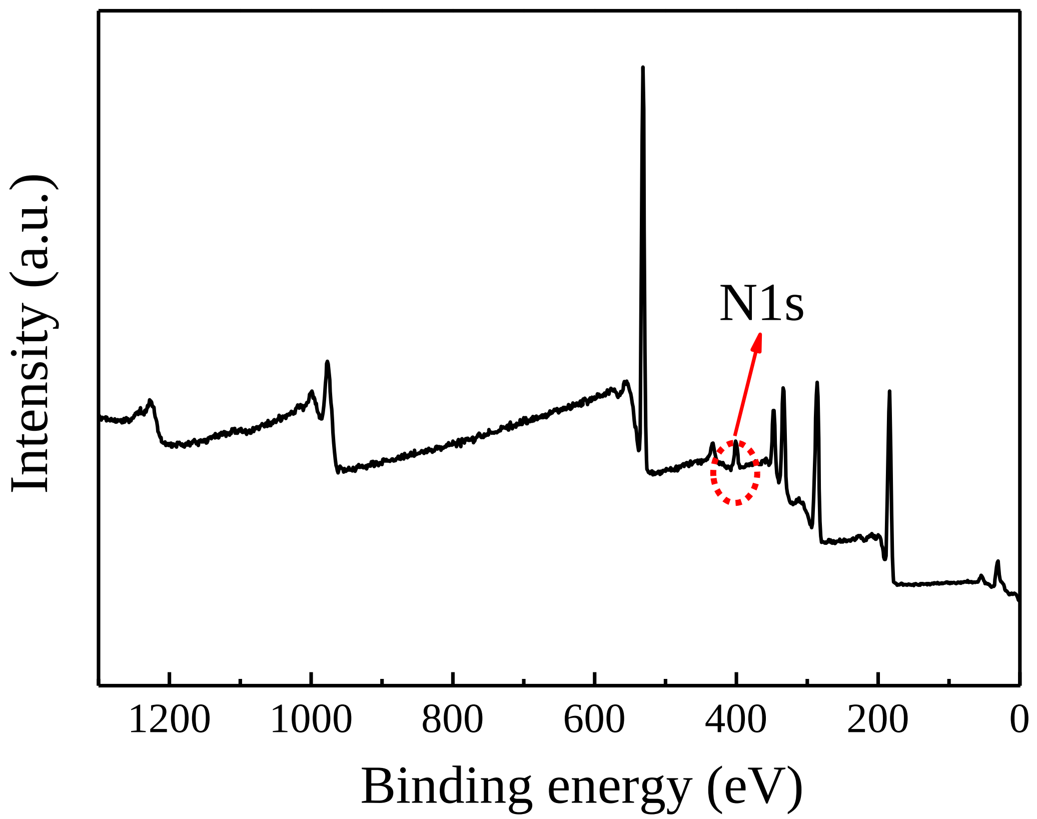

Supplementary Figure 3 | Wide-scan XPS spectra of MOF-808-EDTA.

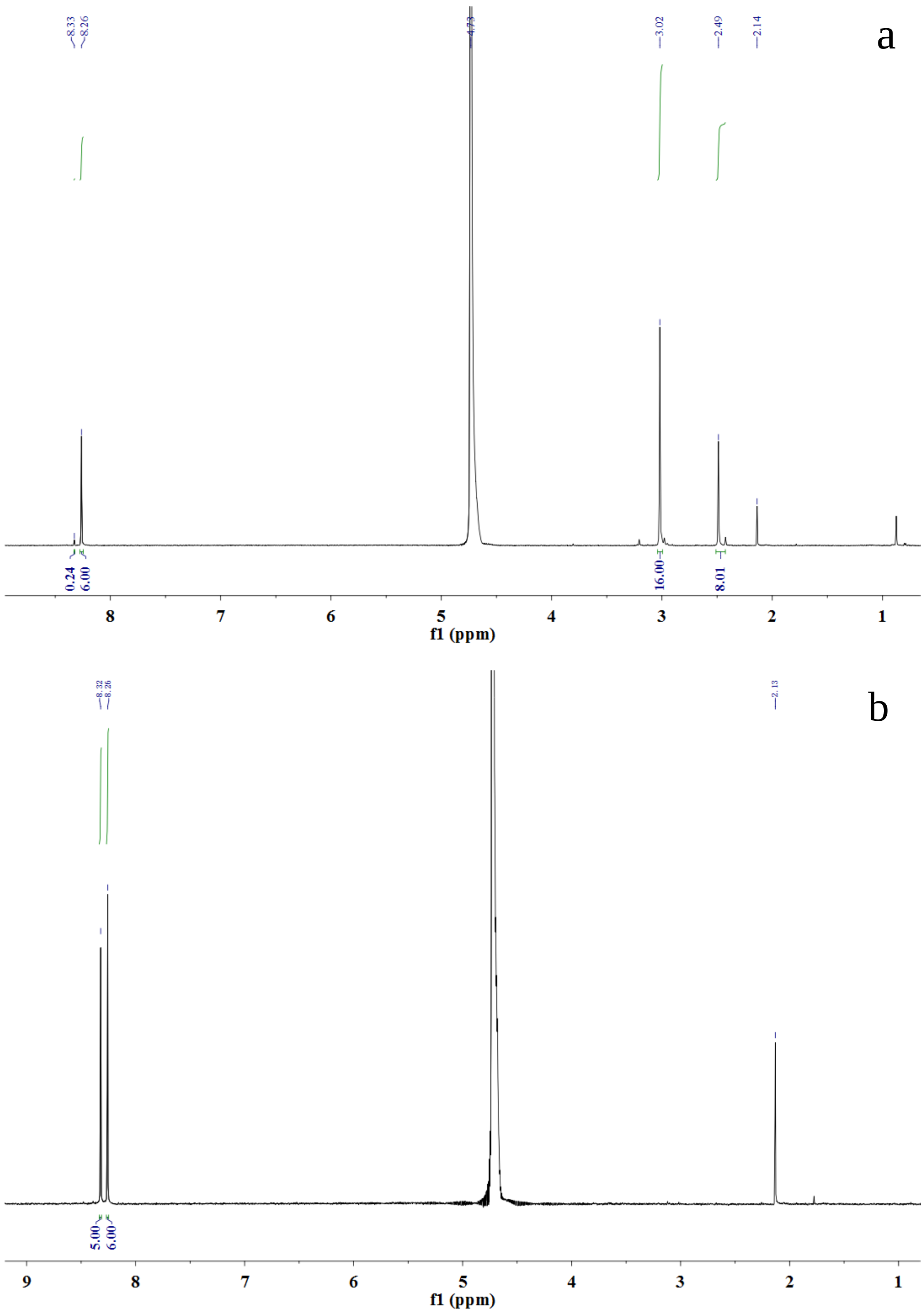

Supplementary Figure 4 | ^1H NMR spectra of alkaline-digested (KOH/D_2O) (a) MOF-808-EDTA and (b) MOF-808 after integral analysis.

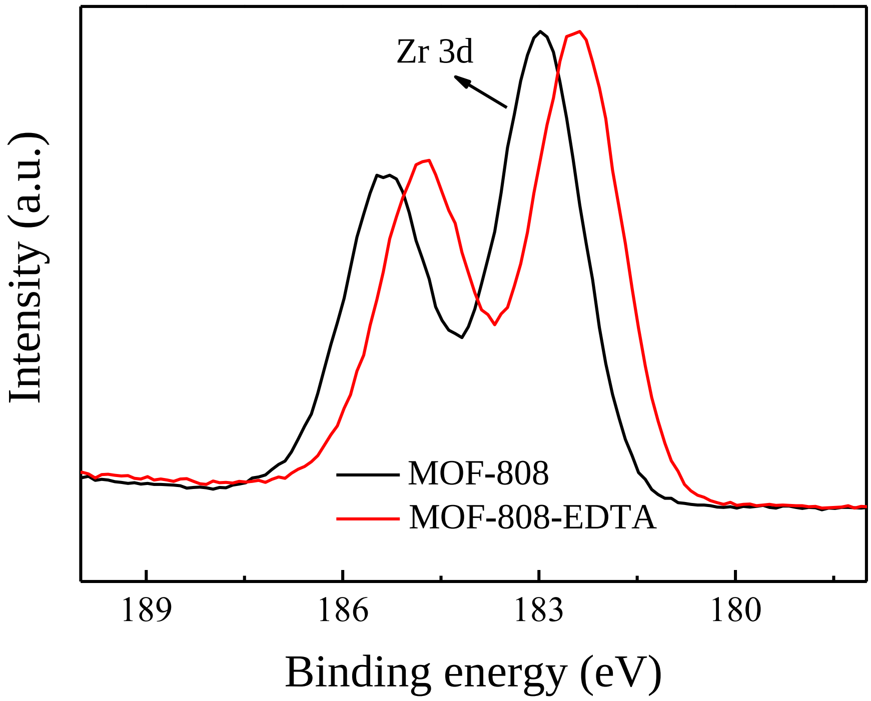

Supplementary Figure 5 | Zr 3d XPS spectra of MOF-808 and MOF-808-EDTA.

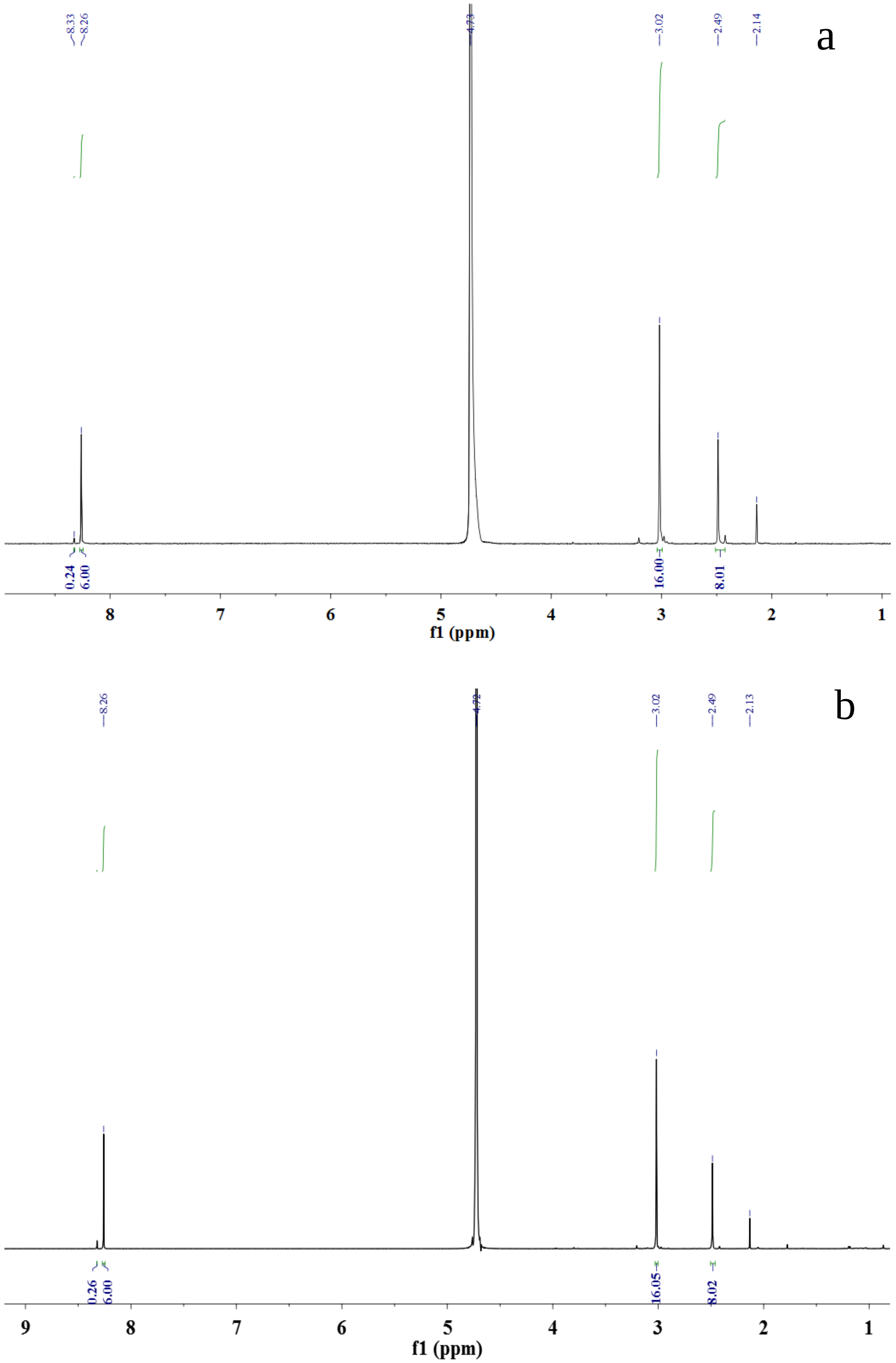

Supplementary Figure 6 | ^{1}H NMR spectra of (a) MOF-808-EDTA and (b) water-washed MOF-808-EDTA after integral analysis.

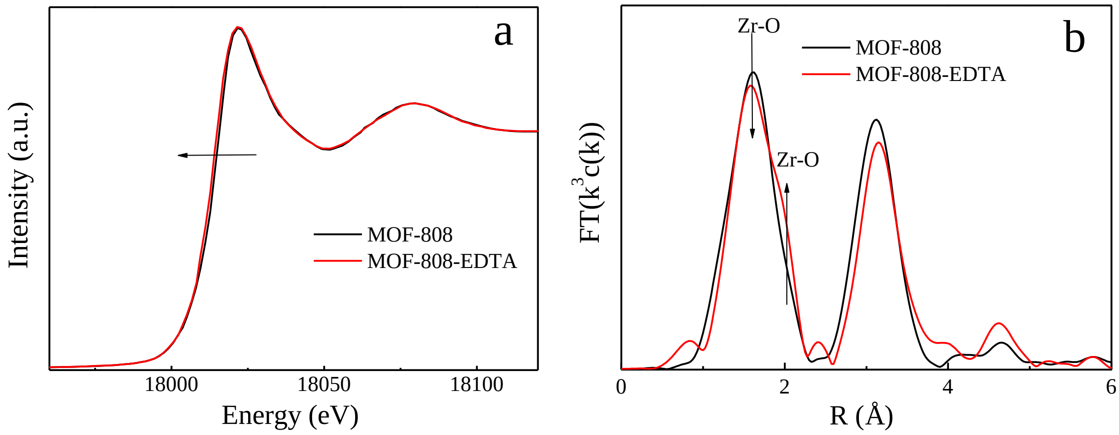

Supplementary Figure 7 | (a) XANES spectra and (b) Zr K-edge EXAFS spectra for Zr_6 cluster in MOF-808 and MOF-808-EDTA.

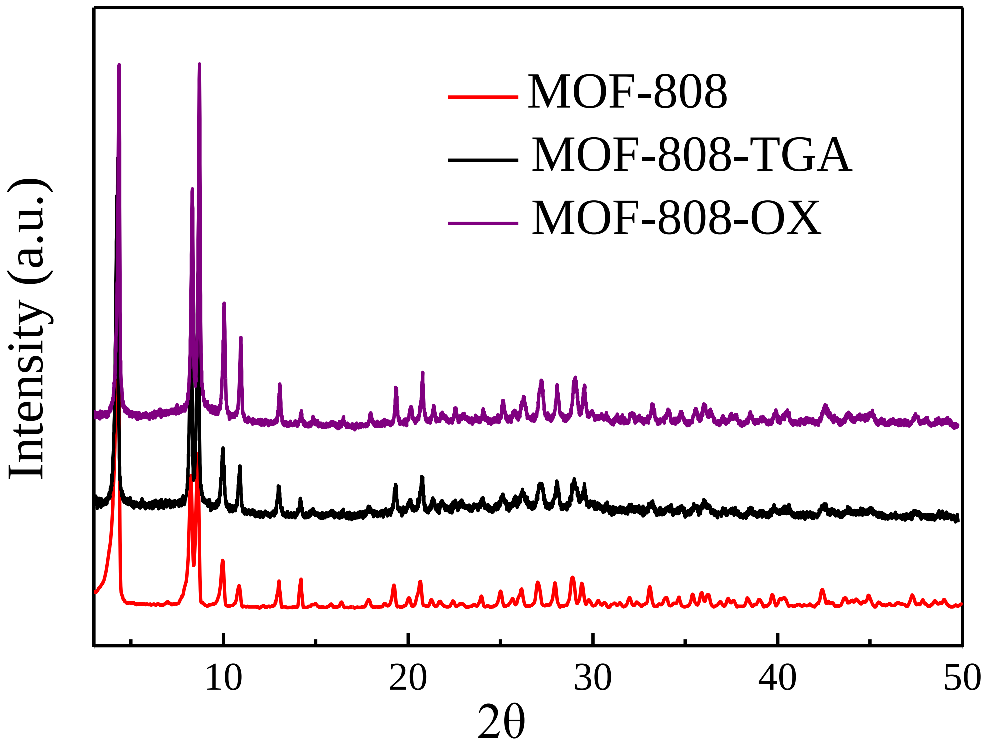

Supplementary Figure 8 | PXRD patterns of MOFs.

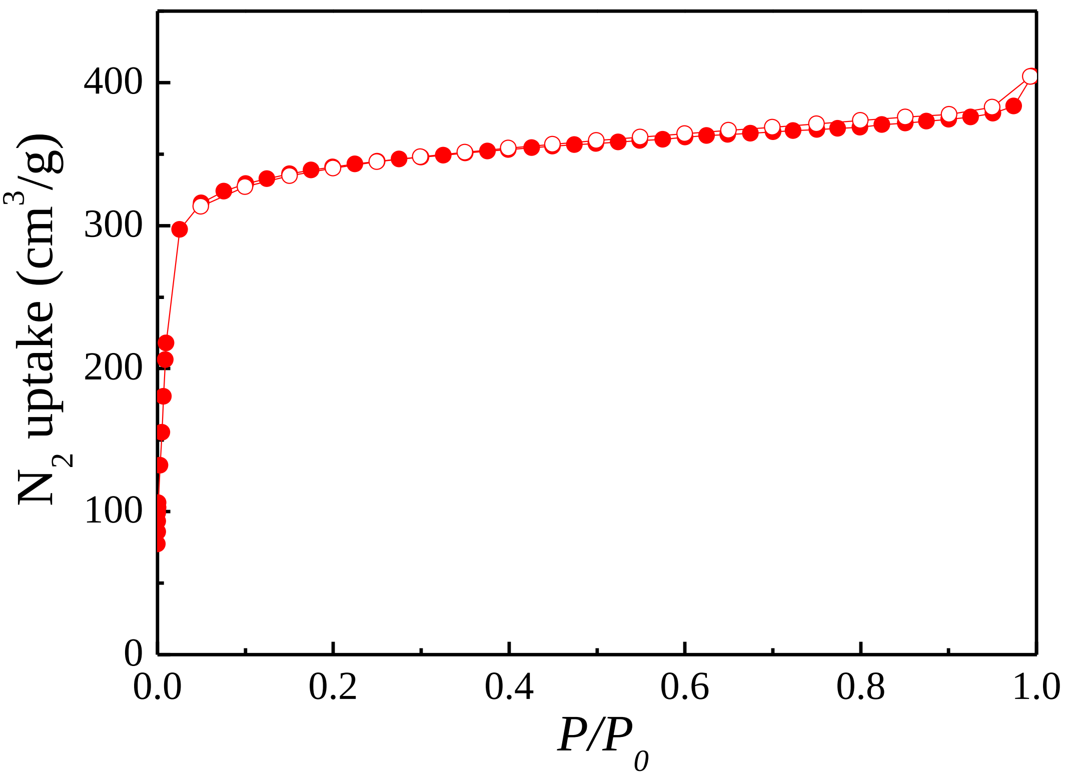

Supplementary Figure 9 | N_2 adsorption-desorption isotherms of MOF-808-TGA at 77 K. The BET surface area was calculated to be 1455 m^2 g^{-1}.

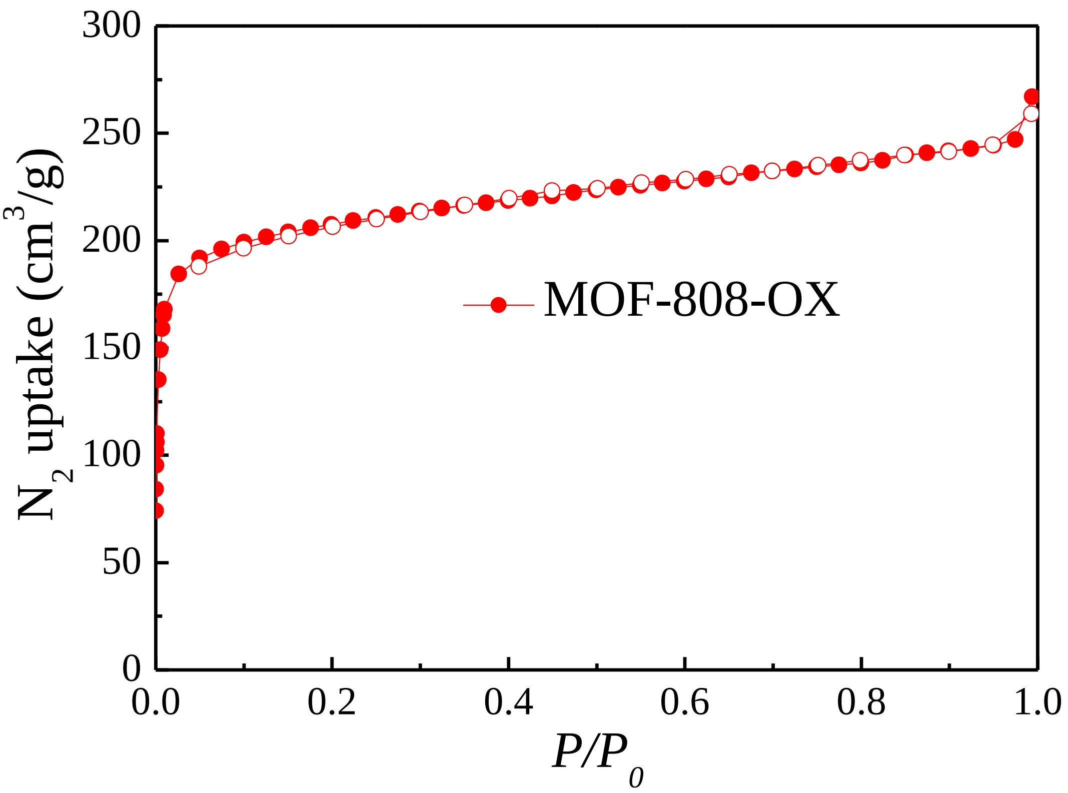

Supplementary Figure 10 | N_2 adsorption-desorption isotherms of MOF-808-OX at 77 K. The BET surface area was calculated to be 813 m^2 g^{-1}.

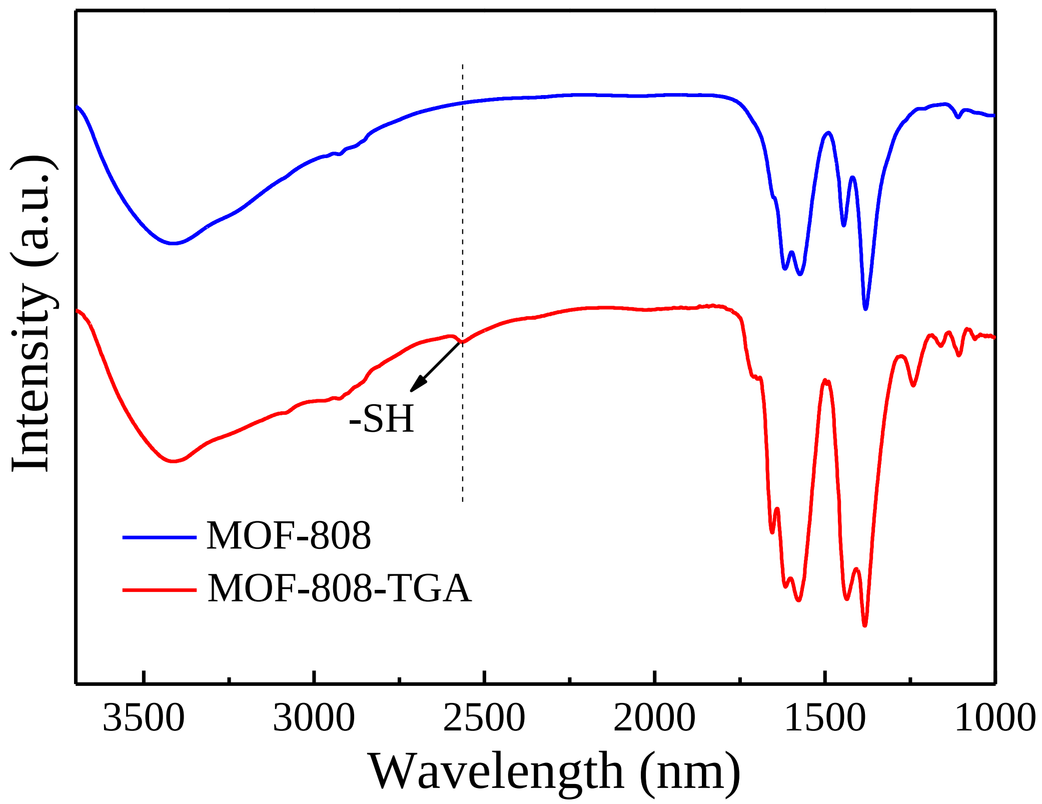

Supplementary Figure 11 | FT-IR spectra of MOF-808-TGA and MOF-808. MOF-808-TGA sample exhibits a new peak at 2564 cm^{-1}, which is consistent with the S-H stretching frequency of free -SH groups.

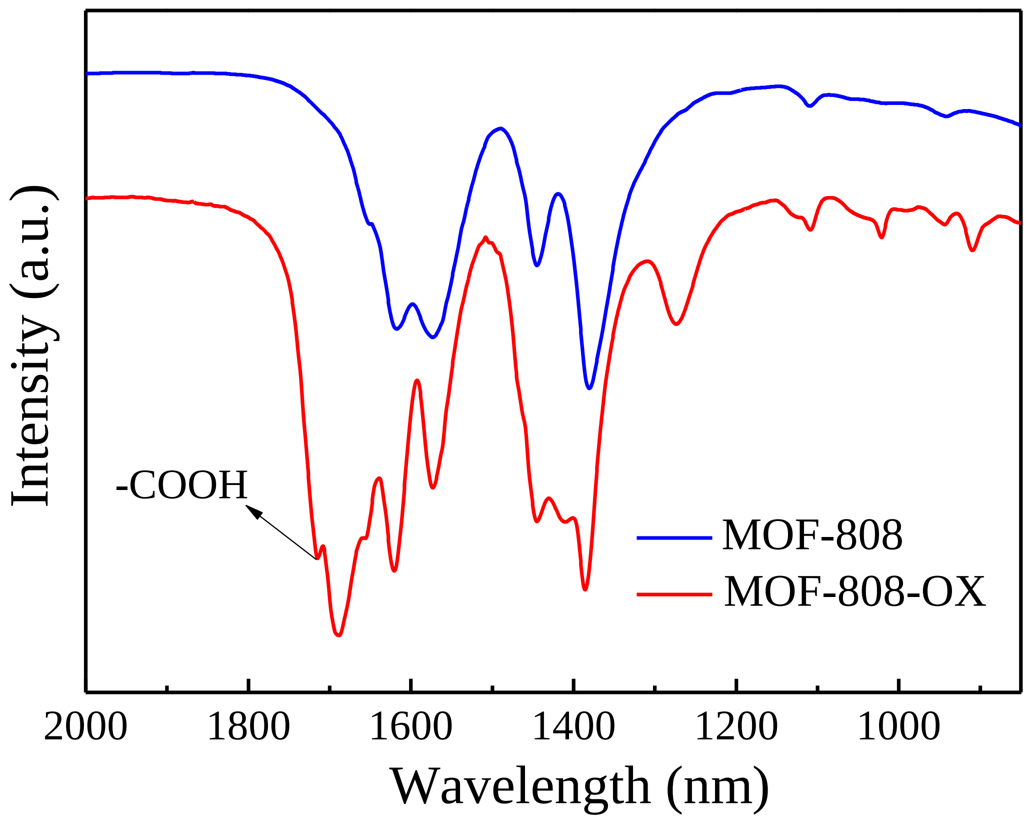

Supplementary Figure 12 | FT-IR spectra of MOF-808-OX (red) and MOF-808 (blue). MOF-808-OX sample exhibits a new peak at 1715 cm^{-1}, which is consistent with the stretching frequency of free -COOH groups.

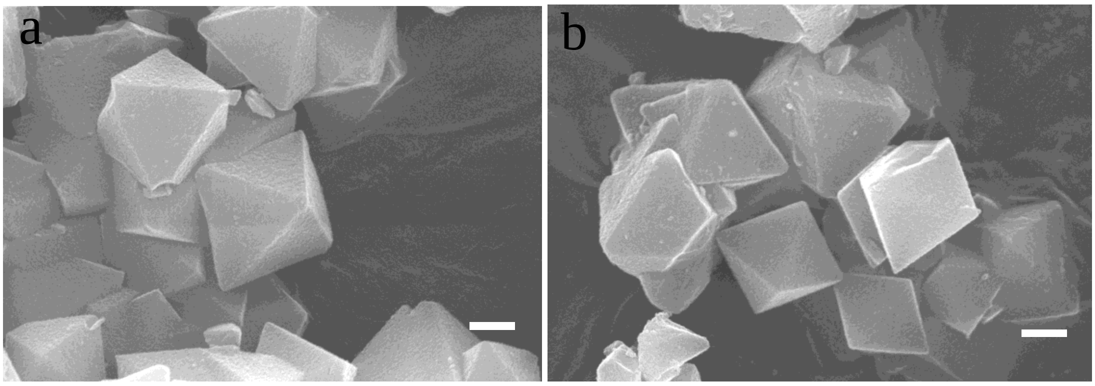

Supplementary Figure 13 | SEM images (Scale bar, 500 nm) of (a) MOF-808-TGA and (b) MOF-808-OX.

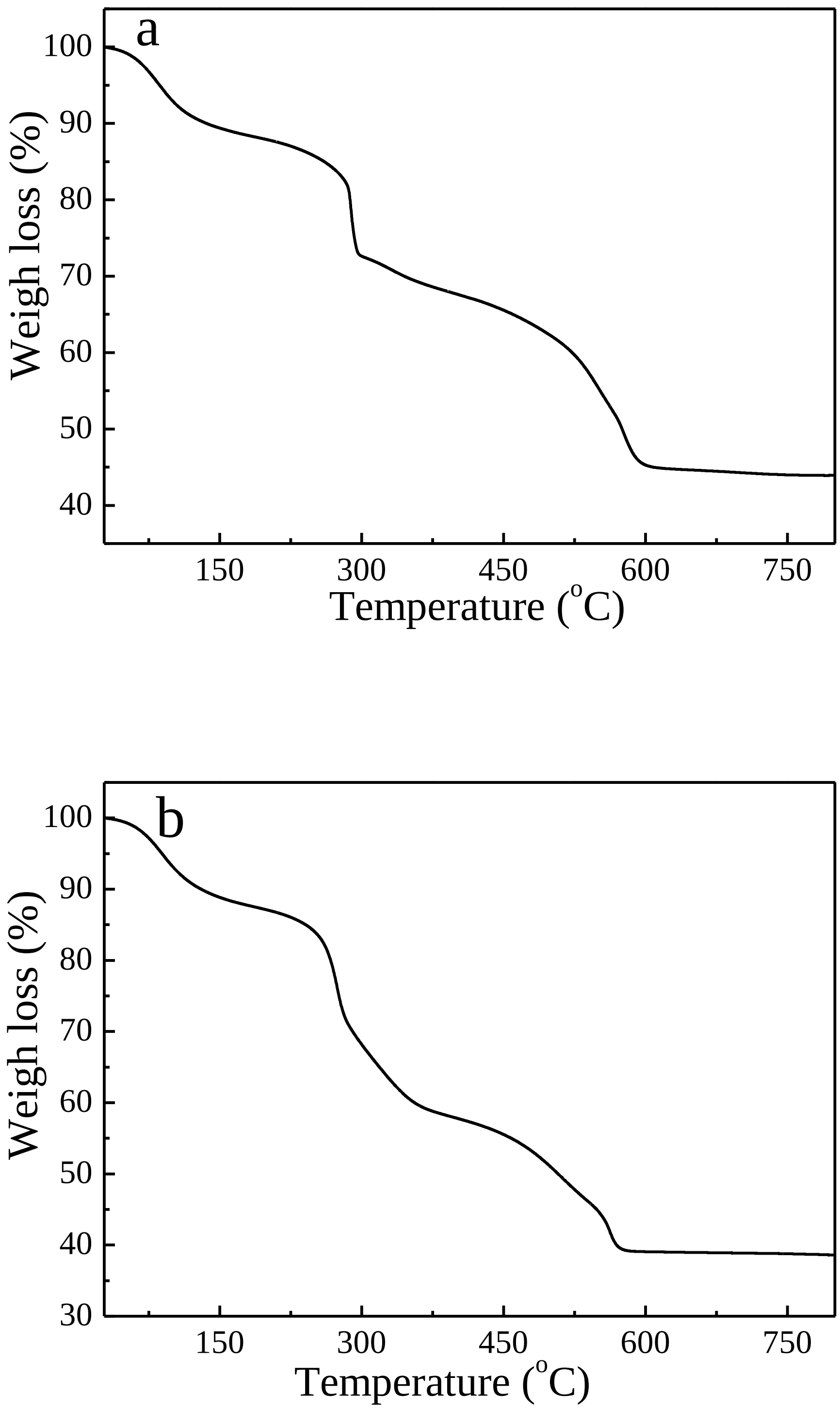

Supplementary Figure 14 | TGA plots of (a) MOF-808-TGA and (b) MOF-808-OX.

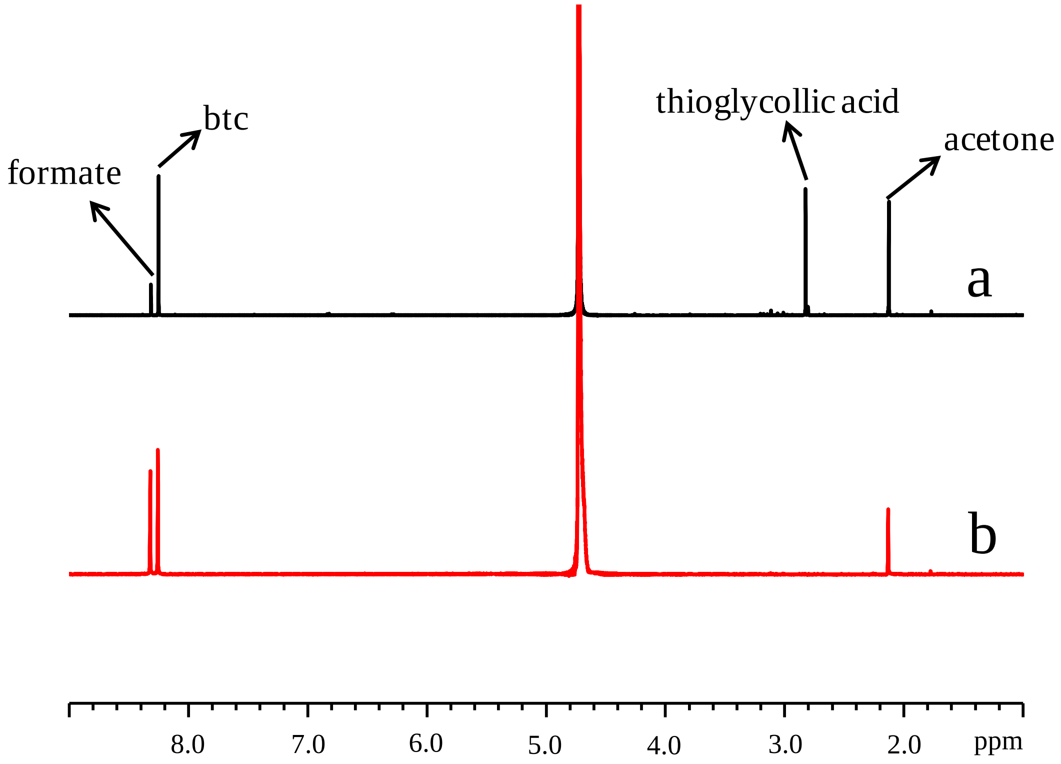

Supplementary Figure 15 | ^{1}H NMR spectra of (a) alkaline-digested MOF-808-TGA and (b) alkaline-digested MOF-808 in KOH/D_2O solution. It is obvious that the intensity of chemical shift at 8.3 ppm for the hydrogen of formate group in MOF-808-TGA reduces significantly and one additional chemical shift at 2.8 ppm corresponding to the hydrogen signal of -CH_2- in thioglycollic acid is present.

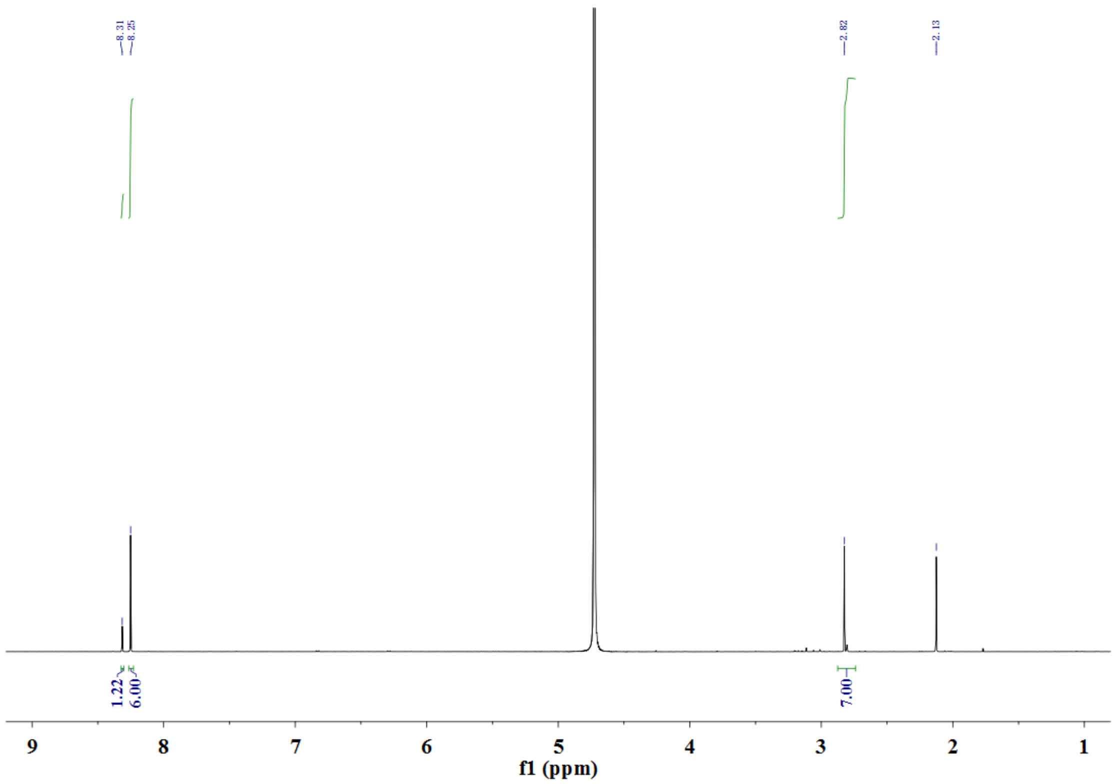

Supplementary Figure 16 | ^{1}H NMR spectra of alkaline-digested (KOH/D_2O) MOF-808-TGA after integral analysis. The two relevant signals of the incorporated thioglycollic acids and residual formate ligands are then integrated against those of btc ligands, resulting peak radios of 6:7 and 6:1.22, respectively.

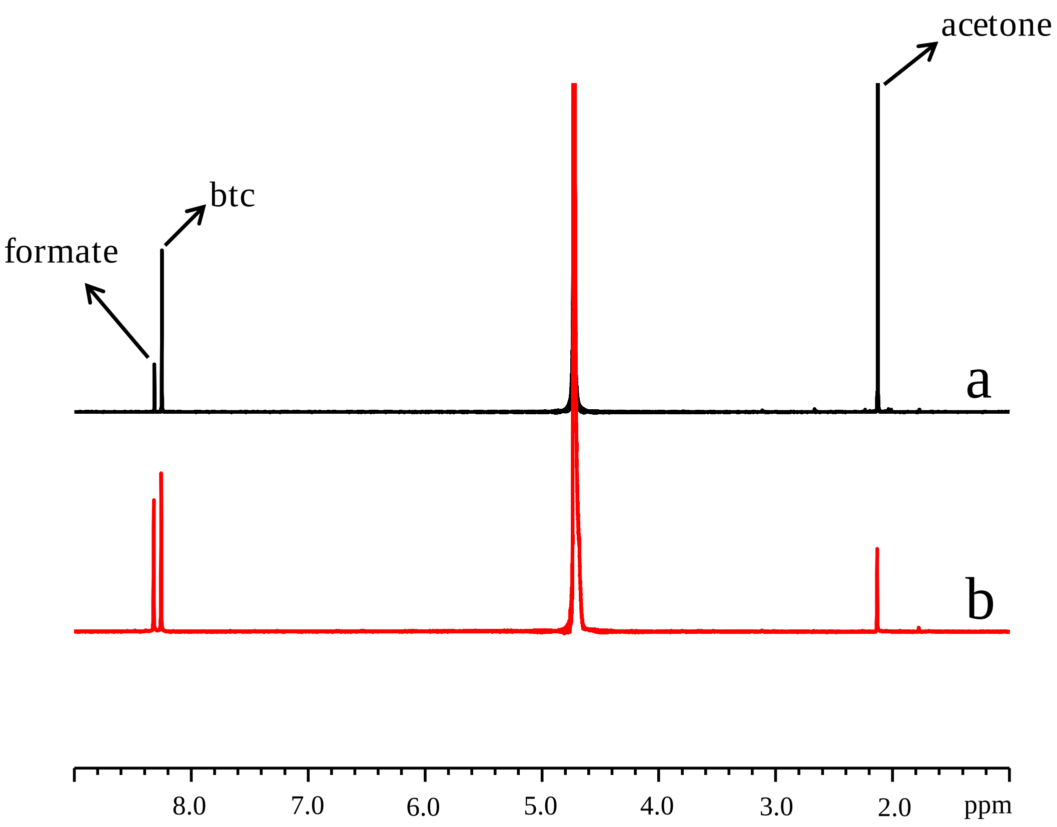

Supplementary Figure 17 | ^{1}H NMR spectra of (a) alkaline-digested MOF-808-OX and (b) alkaline-digested MOF-808 in KOH/D_2O solution. It is obvious that the intensity of chemical shift at 8.3 ppm for the hydrogen of formate group in MOF-808-OX reduces significantly.

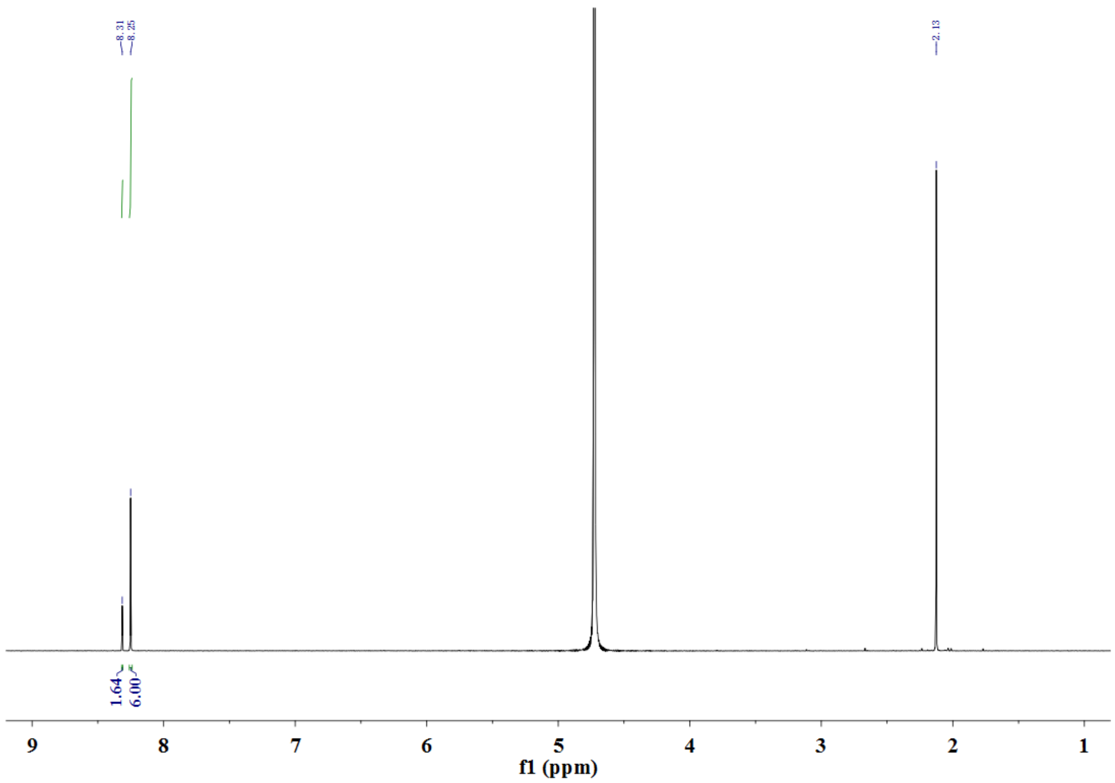

Supplementary Figure 18 | ^1H NMR spectra of alkaline-digested (KOH/D_2O) MOF-808-OX after integral analysis. The relevant signals of residual formate ligands are then integrated against those of btc ligands, resulting in peak radios of 6:1.64.

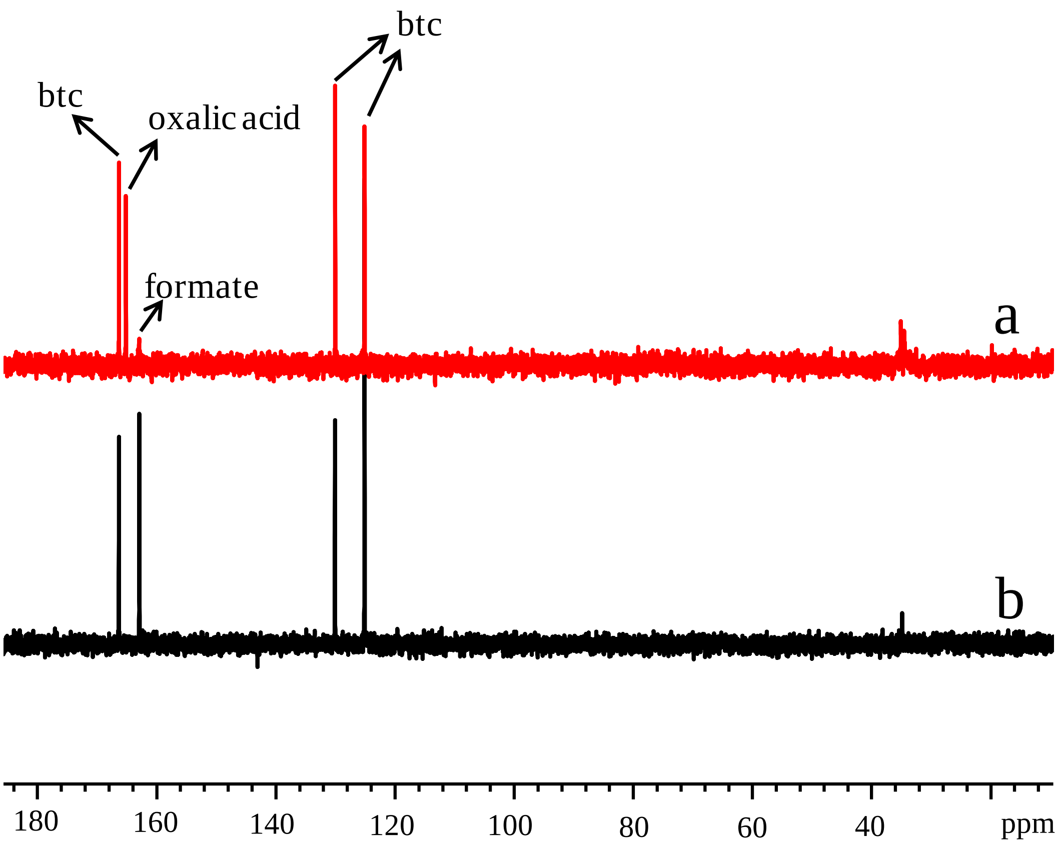

Supplementary Figure 19 | ^{13}C NMR spectra of (a) alkaline-digested MOF-808-OX and (b) alkaline-digested MOF-808 in KOH/D_2O solution. It is obvious that the intensity of chemical shift at 171.1 ppm for the carbon of formate group in MOF-808-TGA reduces significantly and one additional chemical shift at 173.4 ppm corresponding to the carbon signal of oxalic acid is present.

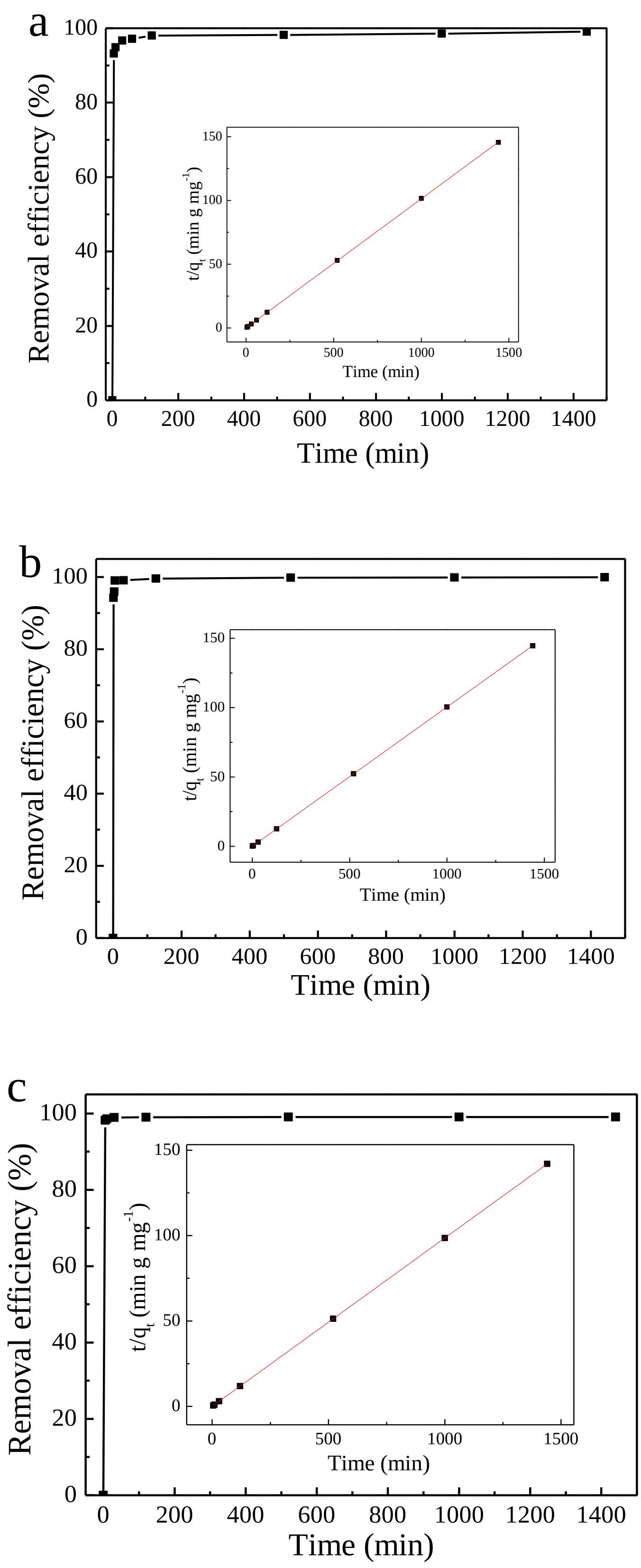

Supplementary Figure 20 | Kinetics investigation of MOF-808-EDTA for metal ions adsorption. (a) La^{3+}, (b) Hg^{2+} and (c) Pb^{2+}. Insets show the pseudo-second-order kinetic plots for the adsorption.

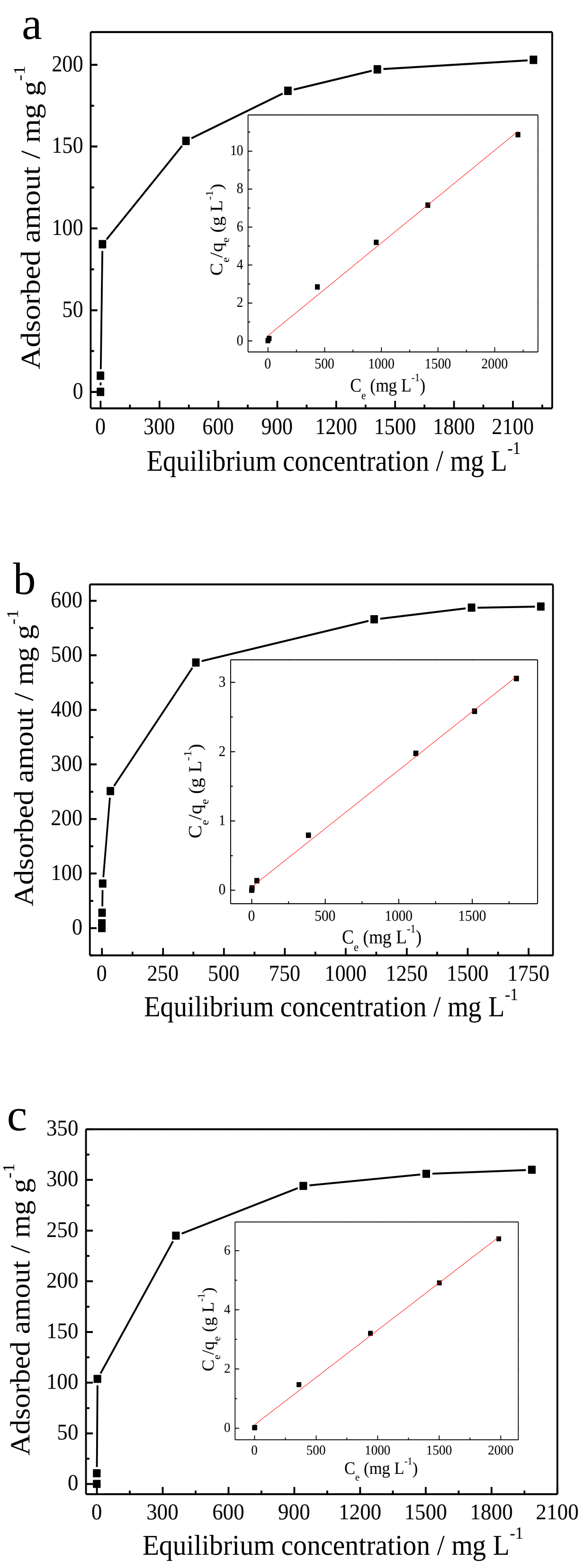

Supplementary Figure 21 | Adsorption isotherms of MOF-808-EDTA for metal ions. (a) La^{3+}, (b) Hg^{2+} and (c) Pb^{2+}. Insets show the linear regressions by fitting the equilibrium adsorption data with Langmuir adsorption model.

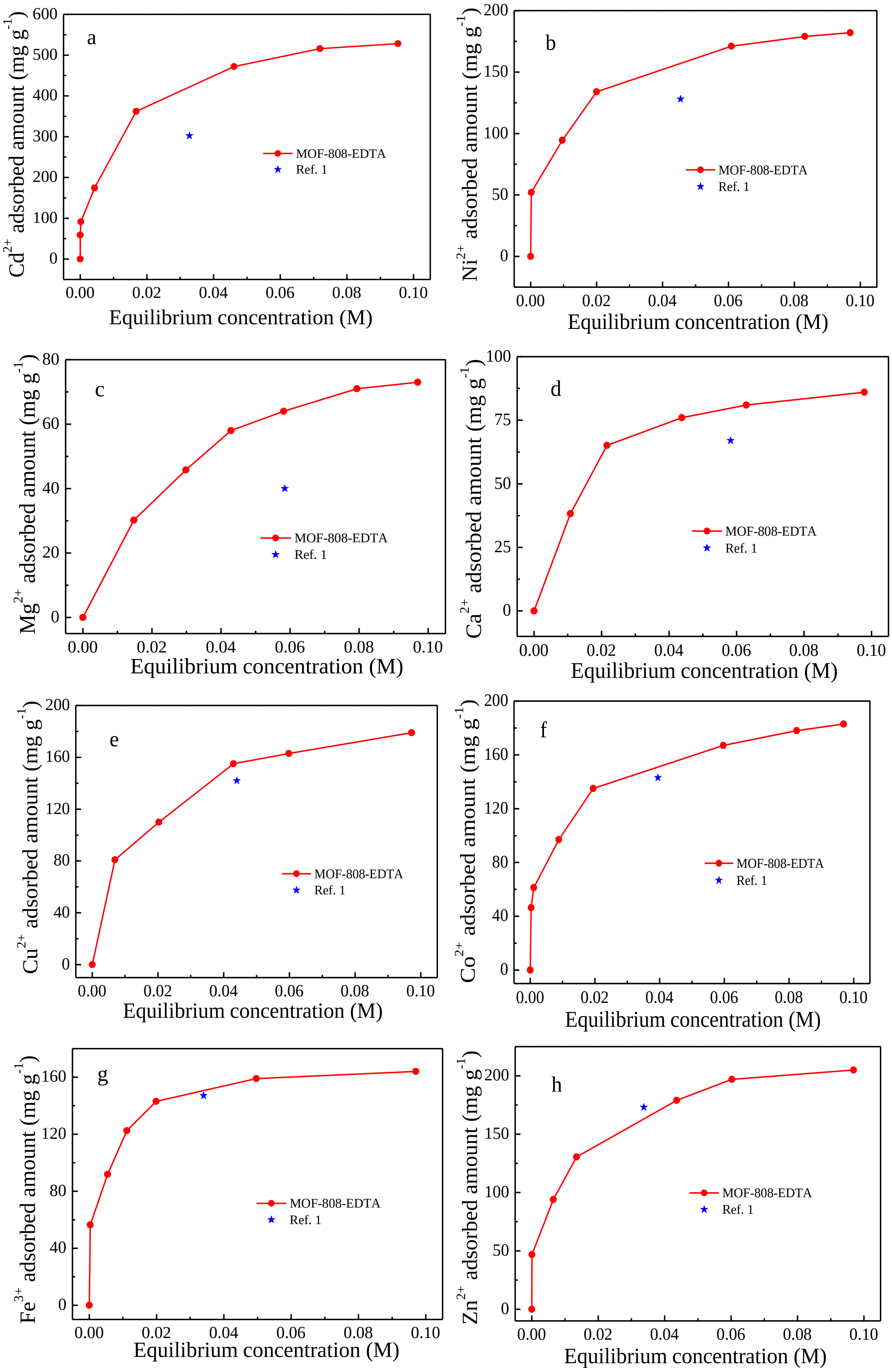

Supplementary Figure 22 | Comparison of adsorption capacity of metal ions in MOF-808-EDTA with these of the organic resin reported in Supplementary Ref 1.

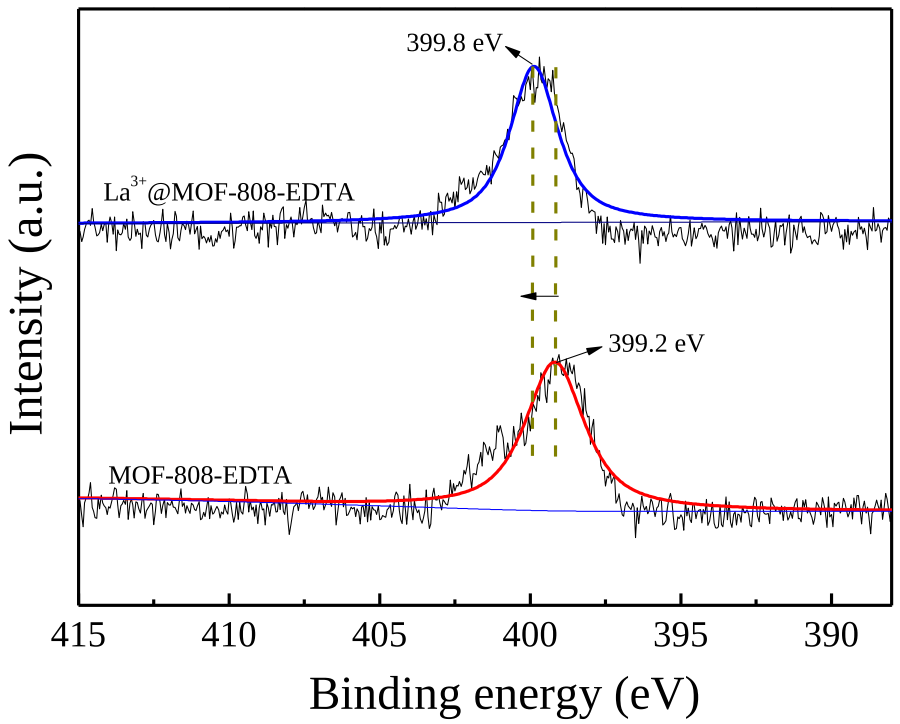

Supplementary Figure 23 | N1s XPS spectra of MOF-808-EDTA before and after La^{3+} loading.

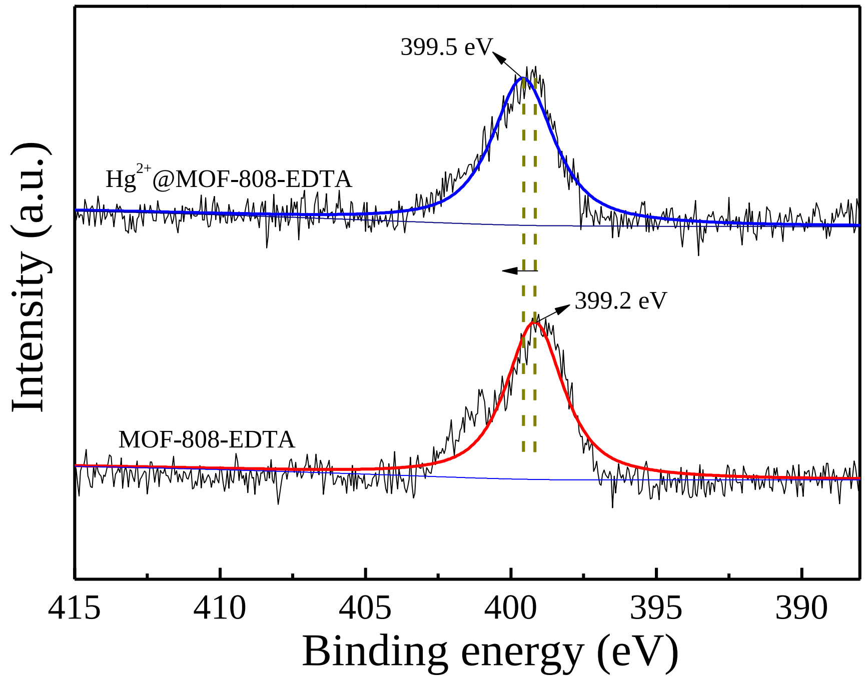

Supplementary Figure 24 | N1s XPS spectra of MOF-808-EDTA before and after Hg^{2+} loading.

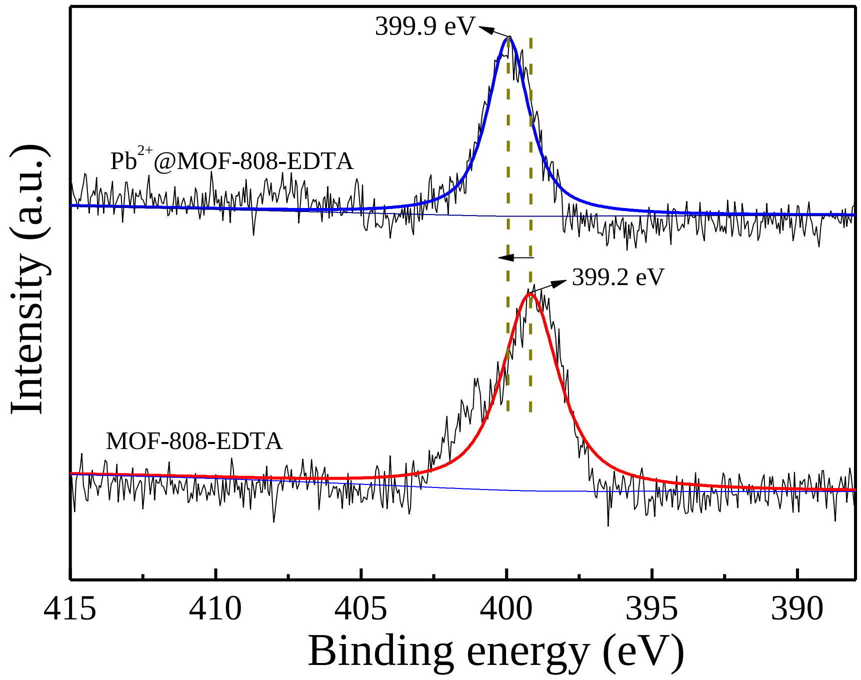

Supplementary Figure 25 | N1s XPS spectra of MOF-808-EDTA before and after Pb^{2+} loading.

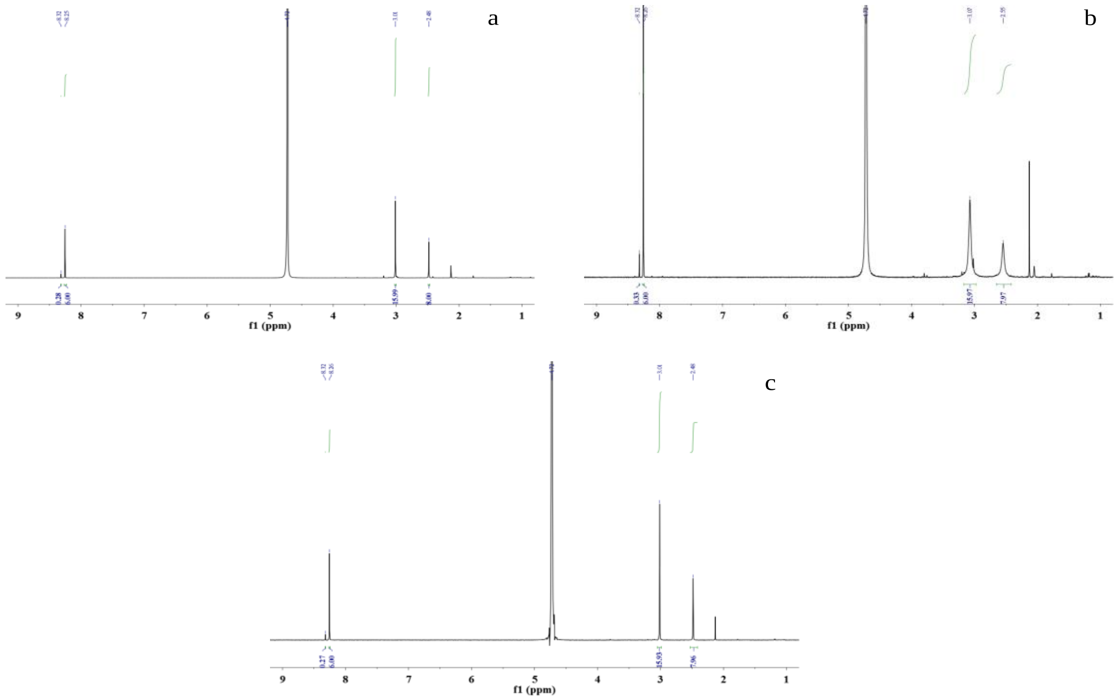

Supplementary Figure 26 | ^{1}H NMR spectra of (a) La^{3+}@MOF-808-EDTA, (b) Hg^{2+}@MOF-808-EDTA and (c) Pb^{2+}@MOF-808-EDTA.

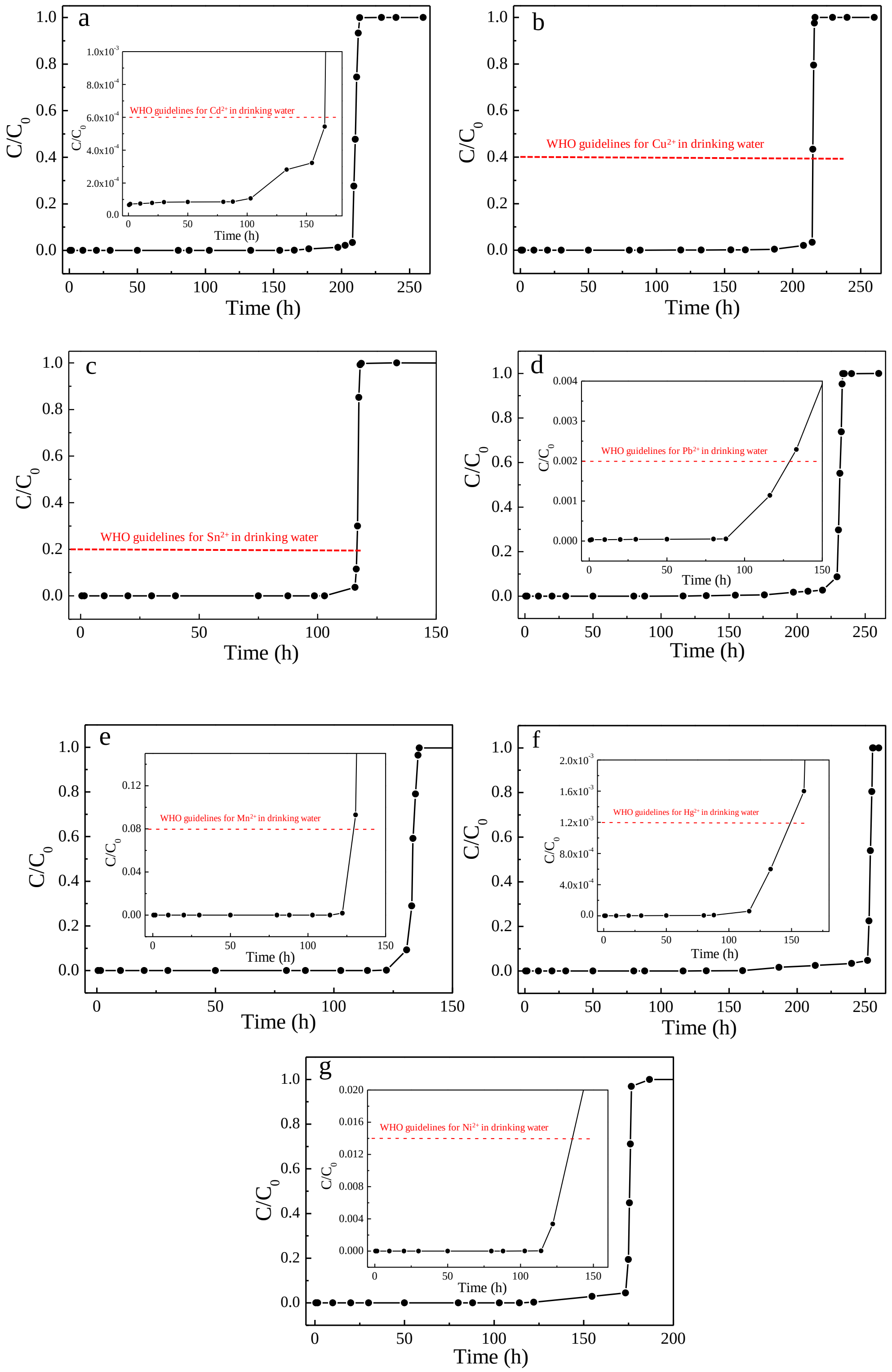

Supplementary Figure 27 | Comparison of the terminal concentrations of metal ions in breakthrough curves with the acceptable limits in drinking water standards of WHO for (a) Cd^{2+}, (b) Cu^{2+}, (c)Sn^{2+}, (d) Pb^{2+}, (e) Mn^{2+}, (f) Hg^{2+}, (g) Ni^{2+}, respectively. Notice: For other metal ions considered in this work, there are no specific guide values in WHO standards.

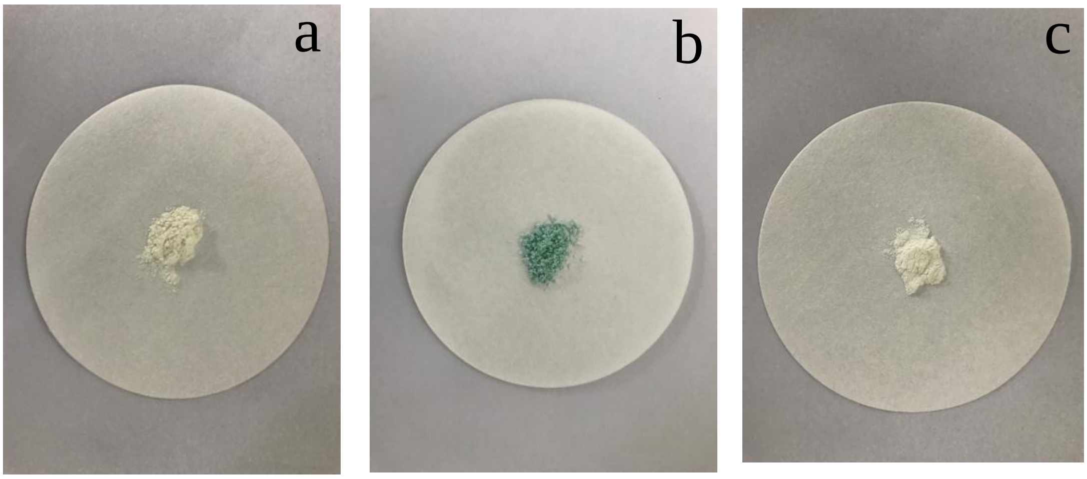

Supplementary Figure 28 | Photographs of (a) MOF-808-EDTA, (b) Cu^{2+}@MOF-808-EDTA and (c) regenerated MOF-808-EDTA.

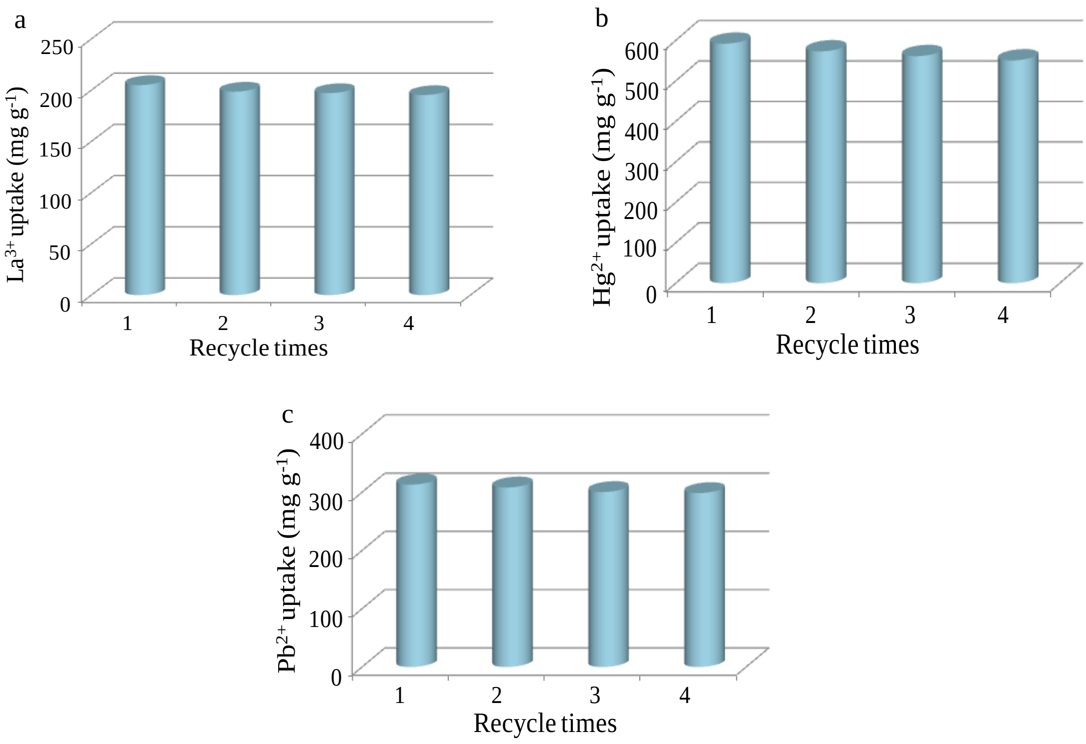

Supplementary Figure 29 | (a) La^{3+}, (b) Hg^{2+} and (c) Pb^{2+} uptake capacities in four cycles.

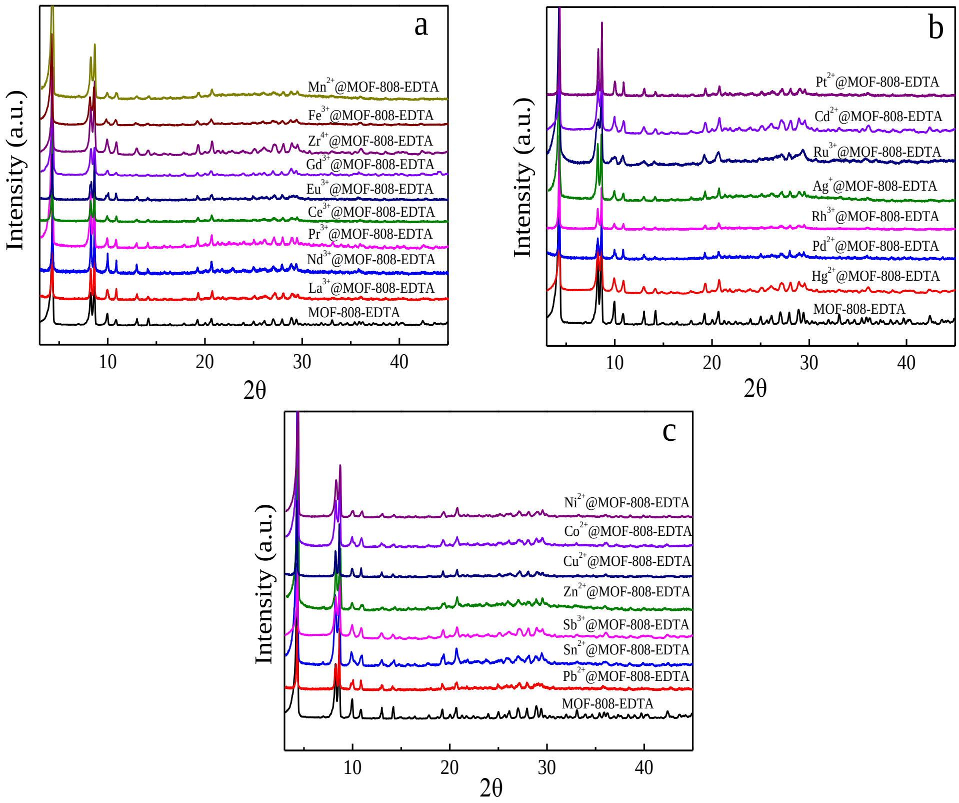

Supplementary Figure 30 | PXRD patterns of MOF-808-EDTA before and after heavy metal ions adsorption: (a) for hard Lewis metal ions, (b) for soft Lewis metal ions, (c) for borderline Lewis metal ions.

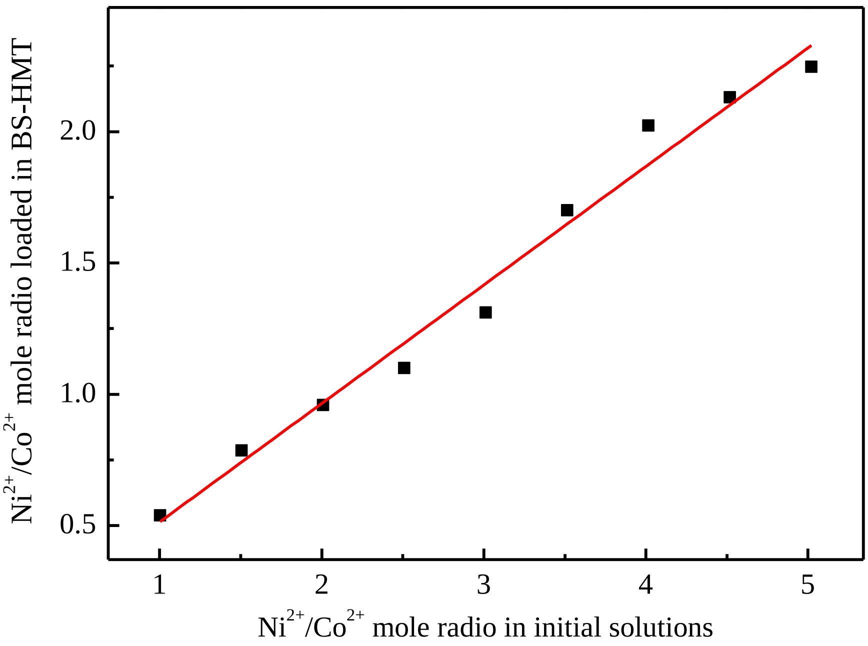

Supplementary Figure 31 | Relationship between the ratio of metal ions loaded in MOF-808-EDTA and initial ratio in solution for Ni^{2+}/Co^{2+}.

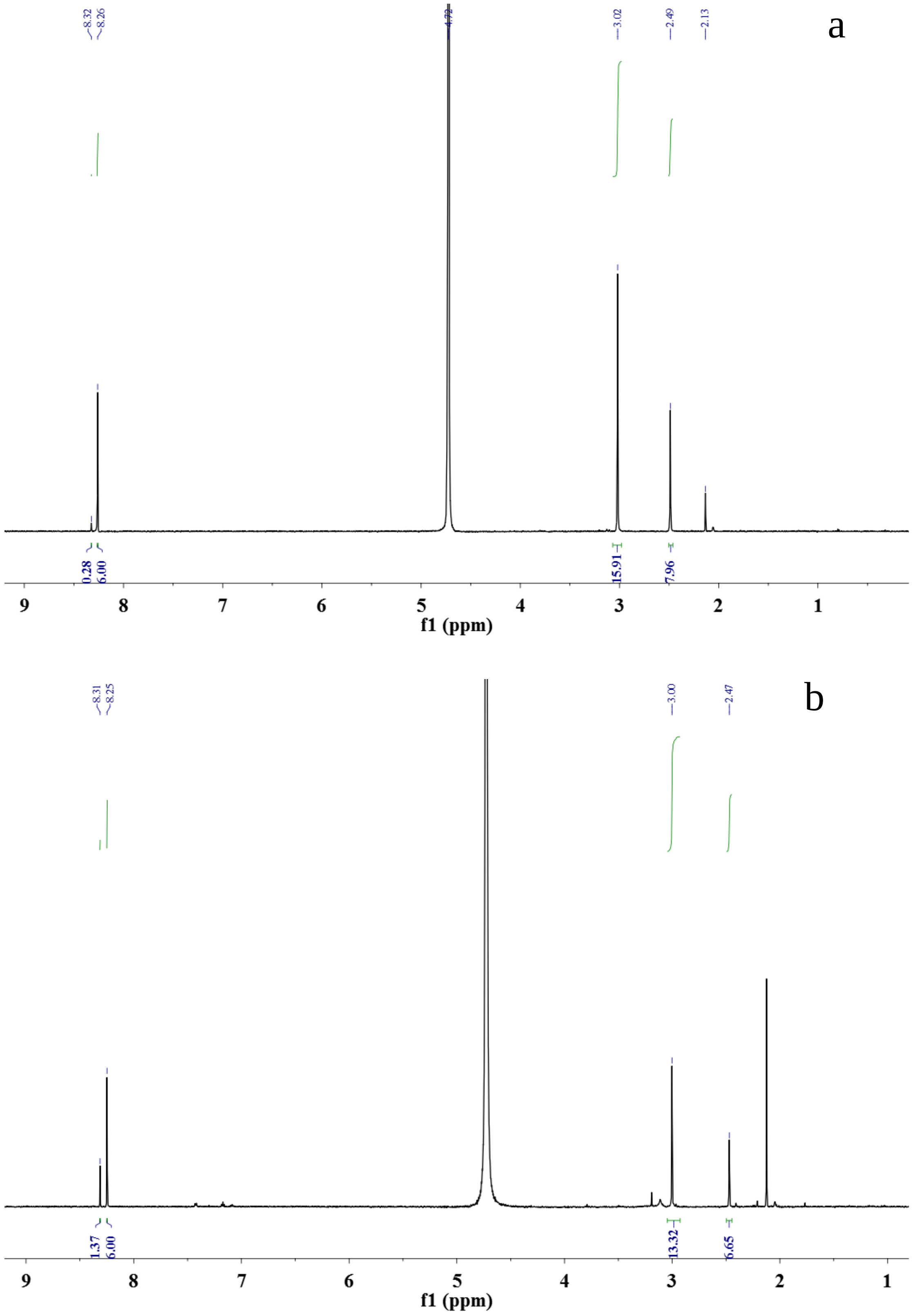

Supplementary Figure 32 | ^{1}H NMR spectra of Pd^{2+}@MOF-808-EDTA (a) before and (b) after Suzuki coupling reaction.

Supplementary Table 1 | Elemental analysis of MOF-808-EDTA before and after being washed with water repeatedly.

	C / %	H / %	N / %
MOF-808-EDTA	25.61	2.26	3.16
MOF-808-EDTA washed with water	25.58	2.28	3.15

Supplementary Table 2 | Comparison of adsorption capacities of MOF-808-EDTA for different metal ions with the organic resin reported in Supplementary Ref 1.

Metal ions	Equilibrium concentration/ mmol L^{-1}	Adsorption capacity of MOF-808-EDTA/ mg g^{-1}	Adsorption capacity reported in Supplementary Ref 1/ mg g^{-1}
Cd^{2+}	33	417	302
Ni^{2+}	46	155	128
Mg^{2+}	59	63	40
Ca^{2+}	58	80	67
Cu^{2+}	44	155	142
Co^{2+}	40	150	143
Fe^{3+}	34	150	147
Zn^{2+}	34	161	173

Supplementary Table 3 | Comparison of adsorption capacities of MOF-808-EDTA for different initial concentrations with the EDTA-like organic resins reported in Supplementary Ref 2.

Metal ions	Initial concentration/ mmol L^{-1}	Molar ratio (adsorbent/metal ion)	Adsorption capacity of MOF-808-EDTA/ mg g^{-1}	Adsorption capacity reported in Supplementary Ref 2/ mg g^{-1}	Removal efficiency of MOF-808-EDTA/ %
Cu^{2+}	0.5	4.1	9.2	54.6	99.9
Ni^{2+}	0.1	5.0	6.9	32.9	99.1
Cd^{2+}	0.1	5.0	13.3	65.3	99.8
Pb^{2+}	0.1	5.0	24.6	107.7	99.9
Co^{2+}	0.05	5.0	6.9	32.9	99.0
Zn^{2+}	0.1	5.0	7.8	37.9	99.8

Supplementary Table 4 | Removal efficiency of Cs^+ for MOF-808-EDTA and the EDTA-like organic resins reported in Supplementary Ref 3.

	MOF-808-EDTA	organic resin reported in Supplementary Ref 3
Removal efficiency [a]/ %	21.03	81.35

[a] The initial Cs^+ concentration is 15 mg L^{-1} .

Supplementary Table 5 | Comparison of the maximum adsorption capacities of MOF-808-EDTA for different metal ions with various EDTA-modified adsorbents including organic and inorganic materials.

Materials	Metal ions	Maximum adsorption capacity/ mg g^{-1}	Supplemen-tary Ref
PVA-EDTA hydrogel	Cd^{2+}	4	4
Si-APTS-EDTA sphere	Cd^{2+}	6	5
Si-APTS-EDTA	Cd^{2+}	24	6
EDTA-Fe_3O_4	Cd^{2+}	49	7
Fe_3O_4@SiO_2-EDTA	Cd^{2+}	51	8
Magnetic EDTA-modified chitosan/SiO_2/Fe_3O_4 adsorbent	Cd^{2+}	63	9
EDTA-modified chitosan-silica hybrid materials	Cd^{2+}	67	10
EDTA functionalized magnetic nanoparticle sorbent	Cd^{2+}	79	11
PS-EDTA	Cd^{2+}	122	12
EDTA-modified cross-linked chitosan	Cd^{2+}	145	13
Magnetic EDTA chitosan adsorbent	Cd^{2+}	169	14
MOF-808-EDTA	Cd^{2+}	528	this work
Si-APTS-EDTA sphere	Pb^{2+}	7	5
PVA-EDTA hydrogel	Pb^{2+}	9	4
EDTA-PCF	Pb^{2+}	26	15
PS-EDTA	Pb^{2+}	32	16
EDTA-Fe_3O_4	Pb^{2+}	99	7
EDTA functionalized magnetic nanoparticle sorbent	Pb^{2+}	100	11

Fe_3O_4@SiO_2-EDTA	Pb^{2+}	114	8
BAC@SiO_2-EDTA	Pb^{2+}	123	17
Magnetic EDTA-modified chitosan/SiO_2/Fe_3O_4 adsorbent	Pb^{2+}	124	9
EDTA-modified chitosan-silica hybrid materials	Pb^{2+}	128	10
PS-EDTA/P	Pb^{2+}	191	18
EDTA-modified biomass	Pb^{2+}	192	19
EDTA-RGO	Pb^{2+}	204	20
Magnetic EDTA chitosan adsorbent	Pb^{2+}	213	14
EDTA-modified Cross-Linked Chitosan	Pb^{2+}	265	13
EDTA-SBA-15	Pb^{2+}	273	21
EDTA-GO	Pb^{2+}	479	20
EDTA-mGO	Pb^{2+}	508	22
MOF-808-EDTA	Pb^{2+}	313	this work
PS-EDTA	Hg^{2+}	218	23
Matal-oxides modified PS-EDTA resin	Hg^{2+}	250	23
EDTA-mGO	Hg^{2+}	268	22
MOF-808-EDTA	Hg^{2+}	592	this work

Supplementary Table 6 | Catalytic performance of Pd^{2+}@MOF-808-EDTA for Suzuki coupling reactions.

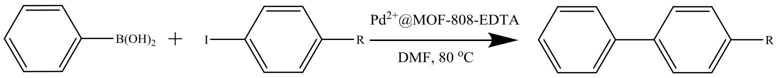

Entry	Substrate	Product	Catalyst	Conversion (%)	Selectivity (%)
1	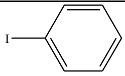	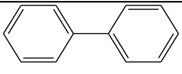	Pd^{2+}@MOF-808-EDTA	99.5	99.8
2	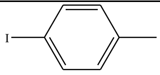	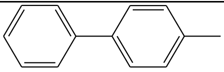	Pd^{2+}@MOF-808-EDTA	>99.9	>99.9
3	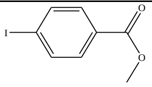	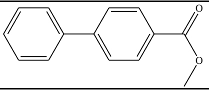	Pd^{2+}@MOF-808-EDTA	>99.9	>99.9
4	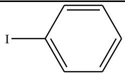	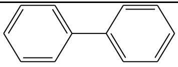	No Catalyst	0.4	0

Supplementary Note 1

In order to get more information for sorption process, the experimental data were fitted with the pseudo-second-order kinetic model using the following equation.

The pseudo-second-order equation:

$$\frac{t}{q_t} = \frac{1}{k_2 {q_e}^2} + \frac{t}{q_e} \qquad \text{(S1)}$$

where q_t (mg g^{-1}) and q_e (mg g^{-1}) are sorption quantity of metal ions at time t (min) and at equilibrium, and k_2 (g $mg^{-1}min^{-1}$) is the rate constant of pseudo-second-order equation. Three extremely high correlation coefficients (> 0.9999) were observed and the values of adsorption rate constants k_2 were calculated to be 0.08 g $mg^{-1}min^{-1}$, 0.41 g $mg^{-1}min^{-1}$, 1.59 g $mg^{-1}min^{-1}$ for La^{3+}, Hg^{2+} and Pb^{2+}, respectively.

Supplementary Note 2

In order to estimate the heavy metal ions uptake capacities of MOF-808-EDTA, the experimental data were fitted with the Langmuir isotherm model using the following equation.

The Langmuir equation:

$$\frac{c_e}{q_e} = \frac{1}{q_m k_L} + \frac{c_e}{q_m} \qquad \text{(S2)}$$

where q_e (mg g^{-1})and c_e (mg L^{-1}) are the amount of metal ions adsorbed on the sorbent and the metal concentrations at equilibrium, q_m (mg g^{-1}) is the maximum sorption capacity at monolayer, and k_L is the Langmuir constant. Three high correlation coefficients (> 0.994) were observed and the values of adsorption rate constants k_L were calculated to be 0.017 g $mg^{-1}min^{-1}$, 0.035 g $mg^{-1}min^{-1}$, 0.028 g $mg^{-1}min^{-1}$ for the adsorption of La^{3+}, Hg^{2+} and Pb^{2+}, respectively.

Supplementary Methods

Synthesis of MOF-808-OX. Activated MOF-808 (0.100 g) and oxalic acid (0.800 g) were immersed in 10 ml DMF solution. The mixture was placed in a 20 ml screw-capped glass jar, which was heated to 60 °C for 24 h. A white precipitate was collected by filtration and washed with DMF and acetone for several times. The resultant solid was then dried at 60 ℃ over night under vacuum condition. Elemental Analysis: C: 22.53%; H: 1.64%; S: 8.07%.

Synthesis of MOF-808-TGA. Activated MOF-808 (0.100 g) and 2 ml of thioglycolic acid were mixed in 10 ml DMF solution. The contents were placed in a 20 ml screw-capped glass jar, which was heated to 60 ℃ for 24 h. A white precipitate was collected by filtration and washed with DMF and acetone for several times. The resultant solid was then dried at 60 ℃ over night under vacuum condition. Elemental Analysis: C: 22.38%; H: 1.17%; N: 0.03%.

Supplementary References

1. Biçak, N., Senkal, B.F. & Melekaslan, D. Poly (styrene sulfonamides) with EDTA-like chelating groups for removal of transition metal ions. *J. Appl. Polym. Sci.* **77**, 2749-2755 (2000).

2. Ferruti, P. et al. L-lysine and EDTA polymer mimics as resins for the quantitative and reversible removal of heavy metal ion water pollutants. *J. Polym. Sci. Part A Polym. Chem.* **50**, 5000-5010 (2012).

3. Arrachart, G. et al. Design and evaluation of chelating resins through EDTA- and DTPA-modified ligands. *Sep. Sci. Technol.* **50**, 1882-1889 (2015).

4. Francis, S. & Varshney, L. Studies on radiation synthesis of PVA/EDTA hydrogels. *Radiat. Phys. Chem.* **74**, 310-316 (2005).

5. Gomes, E.C.C. et al. Synthesis of bifunctional mesoporous silica spheres as potential adsorbent for ions in solution. *Chem. Eng. J.* **214**, 27-33 (2013).

6. Melo, D.Q. et al. Adsorption equilibria of Cu^{2+}, Zn^{2+}, and Cd^{2+} on EDTA-functionalized silica spheres. *J. Chem. Eng. Data* **58**, 798-806 (2013).

7. Xu, M. et al. Study on the adsorption of Ca^{2+}, Cd^{2+} and Pb^{2+} by magnetic Fe_3O_4 yeast treated with EDTA dianhydride. *Chem. Eng. J.* **168**, 737-745 (2011).

8. Liu, Y. et al. Multifunctional nanocomposites Fe_3O_4@SiO_2-EDTA for Pb(II) and Cu(II) removal from aqueous solutions. *Appl. Surf. Sci.* **369**, 267-276 (2016).

9. Ren, Y., Abbood, H.A., He, F., Peng, H. & Huang, K. Magnetic EDTA-modified chitosan/SiO2/Fe3O4 adsorbent: Preparation, characterization, and application in heavy metal adsorption. *Chem. Eng. J.* **226**, 300-311 (2013).

10. Repo, E., Warchoł, J.K., Bhatnagar, A. & Sillanpää, M. Heavy metals adsorption by novel EDTA-modified chitosan-silica hybrid materials. *J. Colloid Interf. Sci.* **358**, 261-267 (2011).

11. Huang, Y. & Keller, A.A. EDTA functionalized magnetic nanoparticle sorbents for cadmium and lead contaminated water treatment. *Water Res.* **80**, 159-168 (2015).

12. He, A., Li, X., Ye, Z., Yang, L. & Li, Y. Efficient removal of Cd^{2+} from aqueous solutions by adsorption on PS-EDTA resins: equilibrium, isotherms, and kinetic studies. *J. Environ. Eng.* **138**, 940 - 948 (2012).

13. Ge, H. & Huang, S. Microwave preparation and adsorption properties of EDTA-modified cross-linked chitosan. *J. Appl. Polym. Sci.* **115**, 514-519 (2010).

14. Zhao, F. et al. Green synthesis of magnetic EDTA- and/or DTPA-cross-linked chitosan adsorbents for highly efficient removal of metals. *Ind. Eng. Chem. Res.* **54**, 1271-1281 (2015).

15. Tanhaei, B., Ayati, A., Bamoharram, F.F. & Sillanpää, M. Magnetic EDTA functionalized preyssler cross linked chitosan nanocomposite for adsorptive removal of Pb(II) ions. *Clean-Soil Air Water* **45**, 1700328 (2017).

16. Wang, L. et al. Study on adsorption mechanism of Pb(II) and Cu(II) in aqueous solution using PS-EDTA resin. *Chem. Eng. J.* **163**, 364-372 (2010).

17. Lv, D. et al. Application of EDTA-functionalized bamboo activated carbon (BAC) for Pb(II) and Cu(II) removal from aqueous solutions. *Appl. Surf. Sci.* **428**, 648-658 (2018).

18. Zhang, Y., Li, X., Zheng, W., Ye, Z. & Li, Y. Enhanced removal of Pb^{2+} from water by adsorption onto phosphoric acid-modified PS-EDTA resin: mechanism and kinetic study. *Desalin. Water Treat.* **51**, 7223-7235 (2013),

19. Yu, J., Tong, M., Sun, X. & Li, B. Enhanced and selective adsorption of Pb^{2+} and Cu^{2+} by EDTAD-modified biomass of baker's yeast. *Bioresource Technol.* **99**, 2588-2593 (2008).

20. Madadrang, C.J. et al. Adsorption behavior of EDTA-graphene oxide for Pb (II) removal. ACS Appl. Mater. Interfaces 4, 1186-1193 (2012).

21. Huang, J. et al. Pb (II) removal from aqueous media by EDTA-modified mesoporous silica SBA-15. *J. Colloid Interf. Sci.* **385**, 137-146 (2012).

22. Cui, L. et al. EDTA functionalized magnetic graphene oxide for removal of Pb(II), Hg(II) and Cu(II) in water treatment: adsorption mechanism and separation property. *Chem. Eng. J.* **281**, 1-10 (2015).

23. Li, X. et al. Efficient removal of Hg(II) by polymer-supported hydrated metal oxides from aqueous solution. *Sep. Sci. Technol.* **47**, 729-741 (2012).
